# High-Throughput Genomic Data Reveal Complex Phylogenetic Relationships in *Stylosanthes* Sw (Leguminosae)

**DOI:** 10.3389/fgene.2021.727314

**Published:** 2021-09-23

**Authors:** Maria Alice Silva Oliveira, Tomáz Nunes, Maria Aparecida Dos Santos, Danyelle Ferreira Gomes, Iara Costa, Brena Van-Lume, Sarah S. Marques Da Silva, Ronaldo Simão Oliveira, Marcelo F. Simon, Gaus S. A. Lima, Danilo Soares Gissi, Cícero Carlos de Souza Almeida, Gustavo Souza, André Marques

**Affiliations:** ^1^ Laboratory of Genetic Resources, Federal University of Alagoas, Arapiraca, Brazil; ^2^ Laboratory of Plant Cytogenetics and Evolution, Federal University of Pernambuco, Recife, Brazil; ^3^ *Campus* Xique Xique, Federal Institute of Education, Science and Technology of Bahia, Xique-Xique, Brazil; ^4^ Embrapa Cenargen, Brasília, Brazil; ^5^ Center of Agronomic Sciences, Federal University of Alagoas, Rio Largo, Brazil; ^6^ Department of Biostatistics, Institute of Biosciences–IBB, Plant Biology, Parasitology and Zoology, São Paulo State University–UNESP, Botucatu, Brazil; ^7^ Department of Chromosome Biology, Max Planck Institute for Plant Breeding Research, Cologne, Germany

**Keywords:** sytlosanthes, allopolyploidy, repetitive DNA, organelle genome, chloroplast, mitochondrion, alignment and assembly free

## Abstract

Allopolyploidy is widely present across plant lineages. Though estimating the correct phylogenetic relationships and origin of allopolyploids may sometimes become a hard task. In the genus *Stylosanthes* Sw. (Leguminosae), an important legume crop, allopolyploidy is a key speciation force. This makes difficult adequate species recognition and breeding efforts on the genus. Based on comparative analysis of nine high-throughput sequencing (HTS) samples, including three allopolyploids (*S*. *capitata* Vogel cv. “Campo Grande,” *S*. *capitata* “RS024” and *S*. *scabra* Vogel) and six diploids (*S*. *hamata* Taub, *S*. *viscosa* (L.) Sw., *S*. *macrocephala* M. B. Ferreira and Sousa Costa, *S*. *guianensis* (Aubl.) Sw., *S*. *pilosa* M. B. Ferreira and Sousa Costa and *S*. *seabrana* B. L. Maass & 't Mannetje) we provide a working pipeline to identify organelle and nuclear genome signatures that allowed us to trace the origin and parental genome recognition of allopolyploids. First, organelle genomes were *de novo* assembled and used to identify maternal genome donors by alignment-based phylogenies and synteny analysis. Second, nuclear-derived reads were subjected to repetitive DNA identification with RepeatExplorer2. Identified repeats were compared based on abundance and presence on diploids in relation to allopolyploids by comparative repeat analysis. Third, reads were extracted and grouped based on the following groups: chloroplast, mitochondrial, satellite DNA, ribosomal DNA, repeat clustered- and total genomic reads. These sets of reads were then subjected to alignment and assembly free phylogenetic analyses and were compared to classical alignment-based phylogenetic methods. Comparative analysis of shared and unique satellite repeats also allowed the tracing of allopolyploid origin in *Stylosanthes*, especially those with high abundance such as the StyloSat1 in the Scabra complex. This satellite was *in situ* mapped in the proximal region of the chromosomes and made it possible to identify its previously proposed parents. Hence, with simple genome skimming data we were able to provide evidence for the recognition of parental genomes and understand genome evolution of two *Stylosanthes* allopolyploids.

## Introduction

High-throughput sequencing (HTS) technologies have recently emerged as a versatile source of sequencing data allowing researchers to rapidly access different aspects of biodiversity based on four main approaches: genome skimming, RAD-Seq, RNA-Seq, and Hyb-Seq (Dodsworth et al., 2019). Of these, the skimming genome stands out for being the sequencing (usually in low coverage) of small random genome fragments (reads) through Next Generation Sequencing (NGS) technologies. The genome skimming analysis allowed the development of several new bioinformatics tools for genomic analysis of non-model organisms, for instance, RepeatExplorer (Novak et al., 2010; Novak et al., 2013). This pipeline has been used to characterize repetitive fractions of genomes, discover new repetitive elements, perform genomic comparative studies, develop probes for fluorescent *in situ* hybridization (Marques et al., 2015; McCann et al., 2020) or characterize distinct subgenomes in allopolyploids (Han et al., 2005; Hemleben et al., 2007).

Recent studies have shown the potential of genome skimming data for phylogenomic studies. Dodsworth et al. (2015) have demonstrated the potential to build phylogenetic topologies based on repeats abundance. This approach has been improved, incorporating the similarities of repeats to the construction of phylogenetic trees (Vitales et al., 2020). Recently developed tools which perform phylogenetic inferences from entire HTS data without the need of alignment or assembly, i.e., alignment and assembly free approaches (Fan et al., 2015; Sarmashghi et al., 2019), just by counting shared and unique k-mers, may allow the use of repeat-derived reads in phylogenetic inferences. On the other hand, genome skimming data also allows the assembly of complete organelle genomes (plastomes and mitogenomes), as well as large tandem repeats as the ribosomal DNA (rDNA) units (Dodsworth et al., 2019). The use of massive alignments of whole chloroplast genomes is frequently the method of choice for establishing phylogenetic relationships in plants, based on usual phylogenetic approaches as Bayesian Inference, Maximum Likelihood, etc. (Guo et al., 2017; Kim et al., 2019; Xie et al., 2020). Organelle inheritance mostly maternal for most plant species (Reboud and Zeyl, 1994; Greiner et al., 2014), makes the sequence of organelle genomes ideal for identifying patterns of maternal genome inheritance in hybrid species (Gastony and Yatskievych, 1992; Jankowiak et al., 2005). Thus, these different genomic and phylogenomic approaches could be important to characterize the origin and evolution of allopolyploid complexes.

The genus *Stylosanthes* Sw. (Leguminosae) belongs to the subfamily Papilionoideae and has a complex systematics, mainly related to the occurrence of natural allopolyploidy (Stace and Edye, 1984; Vanni, 2017). The taxonomy of the genus remains unsettled and controversial, with various authors favoring between 25 and 42 species, with at least 40 additional synonyms (Cameron and Chakraborty, 2004). At least 16 taxa are thought to have been originated by allopolyploidy, which seems to be directly related to the unresolved taxonomy of the genus (Liu and Musial, 2001). *Stylosanthes* shows close evolutionary proximity with the peanut genus *Arachis* L., forming sister lineages in the clade *Pterocarpus* (tribe Dalbergieae) (Cardoso et al., 2013). The genus is the most economically important forage legumes, with species grown worldwide as a pasture crop with grasses, as well as for land reclamation and restoration, soil stabilization, and regeneration, particularly in regions with low precipitation (Cameron and Chakraborty, 2004).

*Stylosanthes* is highly diversified and morphologically polymorphic, having cultivated pantropical species, mostly described for the American continent with two centers of diversification: Mexico and Brazil. Being the latter the main center of origin and diversification for the genus, with more than 30 species, of which 12 are endemic (Stace and Edye, 1984; da Costa and Valls, 2010; Santos-Garcia et al., 2012; Vanni, 2017). Species circumscription and identification are complex in *Stylosanthes* since many different species have overlapping morphological characters, many of them dubious e/or homoplastic (Costa and Ferreira, 1984; Mannetje, 1984; Vanni, 2017). This makes it necessary to use additional data to taxonomy, such as molecular markers (Liu et al., 1999; Liu and Musial, 2001), molecular phylogeny (Vander Stappen et al., 1999; Vander Stappen et al., 2002), genomics or cytogenetics (Marques et al., 2018; Franco et al., 2020).

The basic chromosome number of *Stylosanthes* is *x* = 10, with occurrence of diploids (2*n* = 20), tetraploids (2*n* = 40) and hexaploids (2*n* = 60) species (Stace and Edye, 1984; Vieira et al., 1993). Studies based on restriction fragment length polymorphisms (RFLP) and sequence-tagged site (STS) analyses identified ten genome compositions in the genus, named A to J (Liu et al., 1999; Ma et al., 2004). However, the origin and evolution of most *Stylosanthes* allopolyploid complexes remain largely unresolved (Maass and Sawkins, 2004). One of the few allopolyploids well characterized from a genomic point of view is *Stylosanthes scabra* Vogel (AABB), a hybrid between species of A and B genomes, i.e. *S*. *hamata* (L.) Taub. or *S*. *seabrana* B.L.Maass & ‘t Mannetje (A genomes) and *S*. *viscosa* (L.) Sw. (B genome) (Marques et al., 2018). This origin was further demonstrated by the whole chloroplast genome versus rDNA phylogeny and genomic *in situ* hybridization (GISH) (Marques et al., 2018). However, the origin of other allopolyploids of agronomic interest such as *S*. *capitata* Vogel (supposed to be a hybrid between species with D and E genomes) is still unknown (Liu et al., 1999; Liu and Musial, 2001; Vander Stappen et al., 2002).

In order to clarify the origin of *Stylosanthes* allopolyploids, we tested the efficiency of different bioinformatic analysis using HTS data from three allopolyploid accessions and six diploids, including five different genome compositions. We focused on the characterization of two allopolyploid complexes: *S*. *scabra* (*S*. *hamata*/*S*. *seabrana + S*. *viscosa*) and *S*. *capitata* (*S*. *macrocephala* M. B. Ferreira and Sousa Costa *+ S*. *pilosa* M. B. Ferreira and Sousa Costa). For this, we performed comparative genomic analysis, anchored by phylogenomic inferences based on whole organelle (plastome and mitogenome), rDNA, satellite DNAs, and total reads. The whole plastome and mitogenome of all these were assembled and characterized comparatively. *In situ* hybridization based on species-specific satDNA repeats has further confirmed the origin of *S*. *scabra* and opens a field for further cytogenetic research on *Stylosanthes* allopolyploids. Finally, based on different phylogenetic approaches we discuss the phylogenetic complexity of the genus and the utility of HTS data to help the characterization of *Stylosanthes* allopolyploids.

## Material and Methods

### Plant Material

Samples of nine *Stylosanthes* species analyzed here, including two complexes (*S*. *scabra* and *S*. *capitata*) are listed in Table 1. For comparative studies, available data from *Arachis hypogaea* L. and our previous data from *S*. *scabra*, *S*. *hamata*, and *S*. *viscosa* (Marques et al., 2018) were used. Plant tissue (young leaves, fresh 5–20 g each) of all nine *Stylosanthes* accessions were collected from plants growing in the greenhouse of the Laboratory of Genetic Resources at Federal University of Alagoas.

**TABLE 1:** List of accessions, ploidy level, reference number, SRA, plastome and mitogenome accession numbers and genome composition.

Specie`s name	Ploidy level/Chromosome number	Code/Accession number	Genbank accession	Plastome accession	Mitogenome accession	Genome composition^a^
*S*. *hamata*	Diploid (2n = 20)	SHA 2701	SRX3517479	NC_039159	MZ747306	A
		LC 7666				
*S*. *viscosa*	Diploid (2n = 20)	SVI 2702	SRX3517481	NC_039161	MZ747307	B
		A-01				
*S*. *scabra*	Tetraploid (2n = 40)	SSC 2703	SRX3517480	NC_039160	MZ747308	AB
		CPAC-5234				
*S*. *capitata*	Tetraploid (2n = 40)	SCA 2705	SRX5395139	MZ747315	MZ747309	AB
		cv. Campo Grande				
*S*. *pilosa*	Diploid (2n = 20)	SPI 2706	SRX5395138	MZ747316	MZ747310	E
		LC 7833				
*S*. *macrocephala*	Diploid (2n = 20)	SMA 2707	SRX5395140	MZ747317	MZ747311	D
		cv. Campo Grande				
*S*. *capitata*	Tetraploid (2n = 40)	SCA 2708	SRR13855961	MZ747318	MZ747312	DE
		RS024				
*S*. *seabrana*	Diploid (2n = 20)	SSE 2709	SRR13855960	MZ747319	MZ747313	A
		LC 6261				
*S*. *guianensis*	Diploid (2n = 20)	SGU 2710	SRR13855959	MZ747320	MZ747314	G
		EPAMIG 906				
*Arachis hypogaea*	Tetraploid (2n = 40)		DRR056349	NC_037358	-	

a Genome compositions following the classification of Liu and Musial. (2001).

### High Throughput Sequencing

The genomic paired-end short reads for *Stylosanthes hamata*, *S*. *viscosa*, and *S*. *scabra* samples, which belong to the *S*. *scabra* complex, were the same obtained by Marques et al. (2018), and downloaded from the available accession numbers on NCBI (Table 1). Similarly, as outgroup in our analyses, we have downloaded genomic paired-end short reads for *Arachis hypogaea*.

For *S*. *pilosa* (LC 7833), *S*. *macrocephala* (cv. Campo Grande), *S*. *capitata* (cv. Campo Grande), *S*. *capitata* (RS024), *S*. *seabrana* (LC 6261), and *S*. *guianensis* (Aubl.) Sw. (EPAMIG 906), we have collected young fresh leaves (±1–5 g) for DNA isolation with the kit NUCLEOSPIN PLANT II (Macherey-Nagel). The isolated DNA was checked in 1% (*p*/v) agarose gel and the concentration measured with a NanoDrop 2000 (Thermo Scientific).

The HTS was done with GenOne Soluções em Biotecnologia, Rio de Janeiro, Brasil, where 5 μg of gDNA were used for each sample for the library preparation. The sequencing library was generated using the NEBNext Ultra II DNA Library Prep Kit for Illumina (New England Biolabs, United Kingdom) following the manufactures recommendations. The genomic DNA was randomly sheared to a final fragmented size of 350 bp by Bioruptor and further selected and ligated with adapters. The library was analyzed for the size distribution of fragments by Agilent2100 Bioanalyzer and quantified by real-time PCR. The library was then paired-end (2 × 150 bp) sequenced with Illumina NovaSeq 6000 sequencer generating 3 Gb per sample.

### De Novo Organelle Genome and rDNA Assembly

Plastomes of *S*. *hamata* (SHA 2701), *S*. *viscosa* (SVI 2702), and *S*. *scabra* (SSC 2703) were already assembled before (Marques et al., 2018). For plastome assembly the total number of unprocessed paired-end reads obtained for *S*. *capitata* (SCA 2705), *S*. *pilosa* (SPI 2706), *S*. *macrocephala* (SMA 2707), *S*. *capitata* (SCA 2708), *S*. *seabrana* (SSE 2709), and *S*. *guianensis* (SGU 2710) were used (Table 1). *De novo* plastome assemblies of reads were performed by NOVOPlasty v3.8.3 (Dierckxsens et al., 2017) using default parameters. As NOVOPlasty does not need quality trimming of the reads, all reads for each species were used. NOVOPlasty was able to assemble a single circularized contig for each species, representing the whole plastome including all regions: Long Single Copy (LSC), Short Single Copy (SSC), and both Inverted Repeats (IRs) regions.

NOVOPlasty v3.8.3 was also used to assemble the mitogenomes from the nine *Stylosanthes* samples. From the nine samples, NOVOPlasty was able to retrieve circular mitogenomes for six species: *S*. *hamata* (SHA 2701), *S*. *viscosa* (SVI 2702), *S*. *scabra* (SSC 2703), *S*. *capitata* (SCA 2705), *S*. *seabrana* (SSE 2709) and *S*. *guianensis* (SGU 2710). For *S*. *capitata* (SCA 2708), *S*. *macrocephala* (SSE 2709), and *S*. *pilosa* (SPI 2706) a single linear contig was obtained. All plastomes and mitogenomes contigs obtained were imported into Geneious v. 9.1.8 and the assembly was checked by mapping the raw reads to the contigs using the Geneious mapper with low sensitivity. Plastomes and mitogenomes were annotated using the Geneious annotation tool, guided by the available Leguminosae plastomes and mitogenomes on NCBI. Annotations were manually checked to correct misannotated regions. Plastome and mitogenome maps were generated using OrganellarGenomeDraw (OGDraw v1.2) (Lohse et al., 2013). Repeats (>95% similarity and >500 bp) in each mitochondrial genome were identified with the “Find Repeats” tool available on Geneious v. 9.1.8. All organelle genome accession numbers are provided in Table 1.

To obtain the complete sequence of 5S and 18S-5.8S-28S (35S) rDNA units including the NTS and ITS spacers, respectively, rDNA contigs from the output of RepeatExplorer2 were identified and used as a reference to map the reads from the entire HTS dataset from each sample. Consensus sequences for both 5S and 35S rDNA units were annotated based on comparison with other rRNA genes in Genbank and used for alignment-based analysis. Alternatively, the reads mapped to each unit were used for the alignment and assembly free approach (see below).

### Synteny Comparison of Mitochondrial Genomes

**FIGURE 1:**
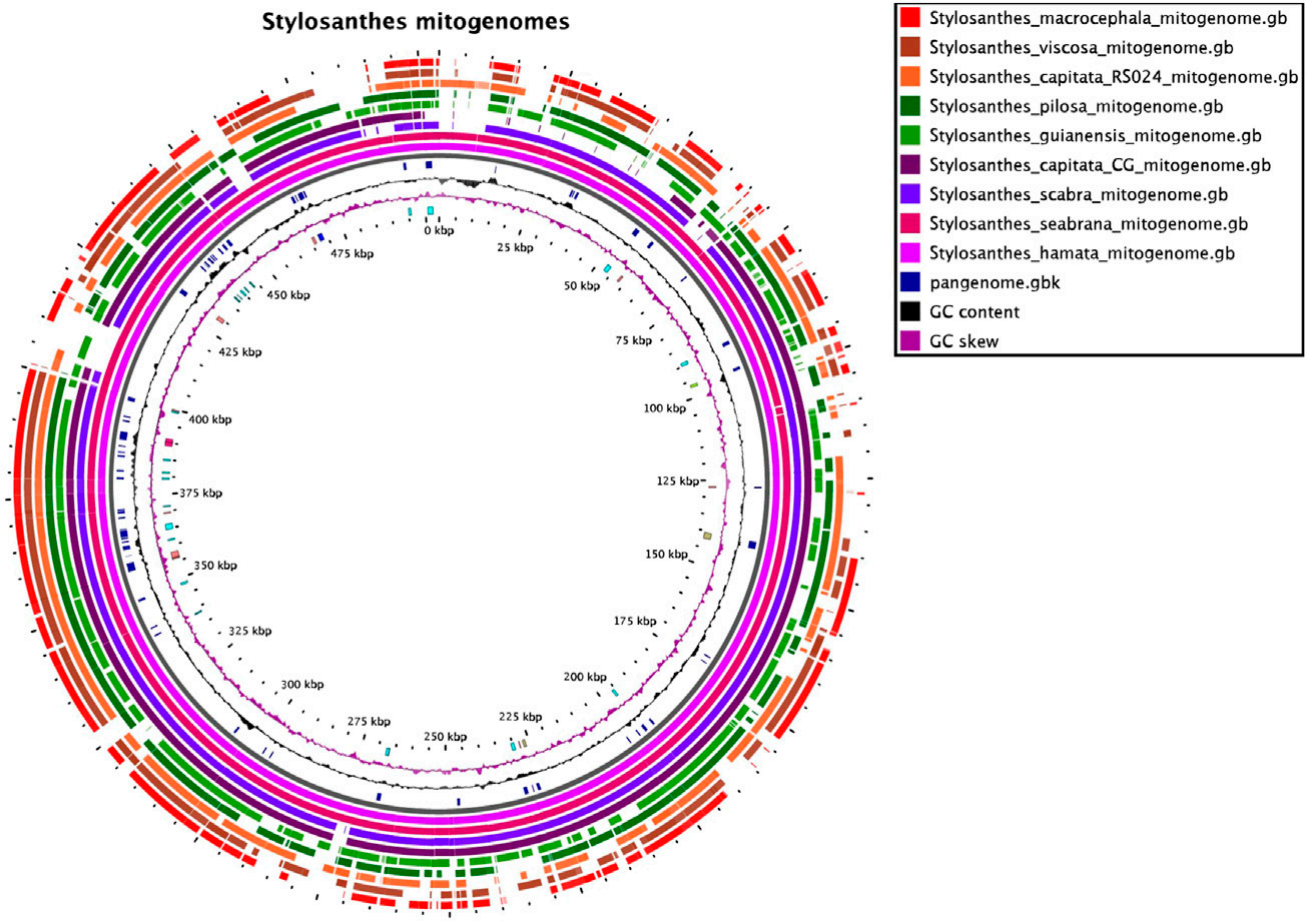
The mitochondrial pangenome of *Stylosanthes*. Annotated sequences and features are shown in the inner circle. For details see Supplementary Figure S1, Supplementary Tables S1, S2.

The nine mitochondrial genomes developed here were compared with each other. The software SyMAP (Soderlund et al., 2011) was used to find syntenic regions in a pairwise-based comparison shown on Figure 1. For this syntenic blocks were calculated based on the annotation and order of genic regions.

### Characterization of DNA Repeats

One million quality filtered paired-end reads of each sample, including *A*. *hypogaea*, were uploaded to the RepeatExplorer2 Galaxy web server (https://repeatexplorer-elixir.cerit-sc.cz/galaxy/) for *de novo* repeat identification and characterization. All samples were subjected to individual and comparative analysis, including *A*. *hypogaea*, using the RepeatExplorer2 (RE2) version with the long run parameters. In comparative mode reads of each species were sampled according to their ploidy levels and genome sizes based on the reference diploid *S*. *hamata* and the polyploid *S*. *scabra* genome sizes (Marques et al., 2018). A custom repeat database was built from the first RE2 run including characterized satDNA repeats from *Stylosanthes* and the repeat library from *A*. *hypogaea* genome (available for download at https://peanutbase.org/) and used for a second run to facilitate repeat annotation and comparison among the samples. Additionally, all samples were also analyzed with TAREAN (Novak et al., 2017), a tandem repeat identification tool available in RE2, which allows quick characterization of sequence composition and diversity of satDNA repeats. Finally, the main repeat clusters were classified into the main repeat families and compared by abundance among the samples.

### Graph-Based Clustering of Satellitome, Mitogenome, and rDNA Reads and Interactive Visualization

Consensus sequences from satDNA repeats identified by TAREAN/RE2, rDNA contigs, and assembled mitogenomes were used as a reference for mapping the entire HTS dataset from each sample. All reads belonging to these three classes of sequences were separately retrieved using Geneious mapper tool with medium-low sensitivity. SatDNA, rDNA, and mitochondrial reads retrieved were separately used as input for comparative graph-based clustering using RepeatExplorer2. Interactive visualization of cluster graphs was performed with the R package SeqGrapheR, which provides a simple graphical user interface for interactive visualization of sequence clusters. SeqGrapheR enabled the selection of species-specific reads from cluster graphs allowing simultaneous viewing of the graph layout (Novak et al., 2010).

### Alignment-Based Phylogenetic Sequence Comparison and Dating

Phylogenetic analyses were initially performed on a subset of diploid samples to avoid the possible uncertain relationships of polyploid specimens in a bifurcating tree. Then, the analysis including the polyploids was conducted using network approaches to account for possible inconsistencies (see below). Alignment of complete plastomes and nuclear 35S rDNA regions were performed with MAFFT v7.222 (Katoh and Standley, 2013) as a Geneious v. 9.1.8 plugin (Kearse et al., 2012). Phylogenetic relationships were inferred using Maximum likelihood with IQ-TREE2 (Nguyen et al., 2015; Minh et al., 2020) and Bayesian Inference (BI) approach implemented in BEAST v.1.8.3 (Drummond et al., 2012). A Markov Chain Monte Carlo (MCMC) run was conducted, sampling every 1,000 generations for 10,000,000 generations using the model GTR. The run was evaluated in Tracer v.1.6 (Rambaut et al., 2014) to determine that the estimated sample size (ESS) for each relevant parameter was >200, and a burn-in of 25% was applied. The majority-rule consensus tree and posterior probability (PP) were visualized and edited in FigTree v.1.4.2 (Rambaut, 2014). Splitstree4 (Huson and Bryant, 2006) was used to generate relationship networks for datasets containing diploids and polyploids, based on the standard function of maximum parsimony. As the outgroup for 1) plastome phylogenetic comparisons, we used the available plastome of *Arachis hypogaea* (KJ468094); 2) rDNA comparisons, we used available ITS1-5.8S-ITS2 regions on NCBI for *Stylosanthes* and *Arachis* species, and 3) 35S rDNA comparisons, the SRA file accession no. DRR056349 from *A*. *hypogaea*, where the assembly of 35S rDNA was performed as described above for *Stylosanthes*.

Divergence time estimates were performed in BEAST v.1.10.4 (Drummond and Rambaut, 2007) fixing the tree topology of the Bayesian analyses. An uncorrelated relaxed lognormal clock (Drummond et al., 2006) and a Yule Process speciation model (Gernhard, 2008) were applied. Two independent runs of 10,000,000 generations each were performed, sampling every 10,000 generations for the full plastome alignment. In order to verify the effective sampling of all parameters and assess the convergence of independent chains, we examined their posterior distributions in Tracer v.1.6, and the MCMC sampling was considered sufficient at an ESS >200. After removing 25% of samples as burn-in, the independent runs were combined and a maximum clade credibility (MCC) tree was constructed using TreeAnnotator v.1.8.2. (Drummond et al., 2012). Calibrations were performed using the secondary calibrations of Särkinen et al. (2012) for the *Arachis*/*Stylosanthes* divergence approx. 12.4 million years ago (Mya).

### Repeat-Based Alignment and Assembly Free Phylogenetic Analysis

To access the phylogenetic signal of diverse repeat class and to avoid loose information we decided to use a recently developed approach that is able to infer phylogenetic trees out of HTS data without the need for alignment using the alignment and assembly free (AAF) tool (Fan et al., 2015). AAF constructs phylogenies directly from unassembled genome sequence data, bypassing both genome assembly and alignment. Using mathematical calculations, models of sequence evolution, and simulated sequencing of published genomes, AAF addresses both evolutionary and sampling issues caused by direct reconstruction, including homoplasy, sequencing errors, and incomplete sequencing coverage. Thus, it calculates the statistical properties of the pairwise distances between genomes, allowing it to optimize parameter selection and perform bootstrapping. Since this approach only needs a set of reads per sample it makes the analysis quite flexible, where we can examine the phylogenetic signal of different sets of sequencing data. Thus, we have made phylogenetic inferences for different sets of data, 1- satellitome reads, which comprises all reads mapped to the consensus satellite DNA of each sample, 2- repeat reads, comprising all reads that were clustered with RE2, 3- all reads, comprising a random subsample from each sample, generated with the *reformat*.*sh* tool (BBMap – Bushnell B. – sourceforge.net/projects/bbmap/)

To check whether satDNA repeats found in the different species are also present in the other species in lower abundance and to identify the different families, we have compiled all consensus sequences from the TAREAN output, which consisted of 54 consensus sequences in total. We have used this file as a reference to uniquely map the total amount of HTS reads from each sample. Mapped reads were grouped by each consensus that they mapped. First, we collected all these reads by species, that were assumed to be a sum of all satellite reads from each sample (satellitome reads). These reads were concatenated in FASTA files and subsequently analyzed in a comparative analysis to test whether identified satDNA repeats are shared among the species and how they group in different clusters. Genomic abundances were then inferred by the number of reads mapped to each satDNA repeat. We have considered only satDNA repeats showing at least 0.01% of genomic abundance in at least one of the samples. For our comparative analysis, we also considered the genomic abundance obtained from our mapping strategy instead of the RepeatExplorer estimations.

### Slide Preparation

For cytogenetic analysis, seeds were germinated and root tips were collected and pretreated with 8-hydroxyquinoline for 20 h at 10°C, fixed in ethanol:acetic acid (3:1; v/v) from 2 to 24 h at room temperature and stored at –20°C. The fixed roots were washed in distilled water and digested in 2% cellulase (Onozuka) and 20% pectinase (Merck) at 37°C for 90 min. Then apical meristems were squashed in 45% acetic acid under a coverslip. The coverslip was removed in liquid nitrogen.

### Probe Labeling and *in situ* Hybridization

In order to localize the satDNA repeats identified in the *S*. *scabra* complex, the repeats were amplified by PCR and labeled with Cy3-dUTP (GE Healthcare) or with digoxigenin-11-dUTP (Merck). All probes were labeled by nick translation (Merck). These labeled probes were used for FISH. Digoxigenin-labeled probes were detected with sheep anti-digoxigenin FITC conjugate (Merck) and amplified with rabbit anti-sheep FITC conjugate (Bio-Rad). FISH was performed according to (Marques et al., 2018). The hybridization mix contained 50% of formamide (v/v), 10% dextran sulfate (w/v), 2 × SSC, and 50 ng of each probe. The final hybridization stringency was estimated to be 76%. The slides were mounted with 4′,6-diamidino-2-phenylindole (DAPI, 4 μg/ml)/Vectashield (Vector) 1:1 (v/v) and analyzed under a Leica epifluorescence microscope and the Leica Las AF software. Overlapping, processing of images for brightness and contrast were performed using Adobe Photoshop® CC 2019.

## Results

### Mitochondrial Genomes

**FIGURE 2:**
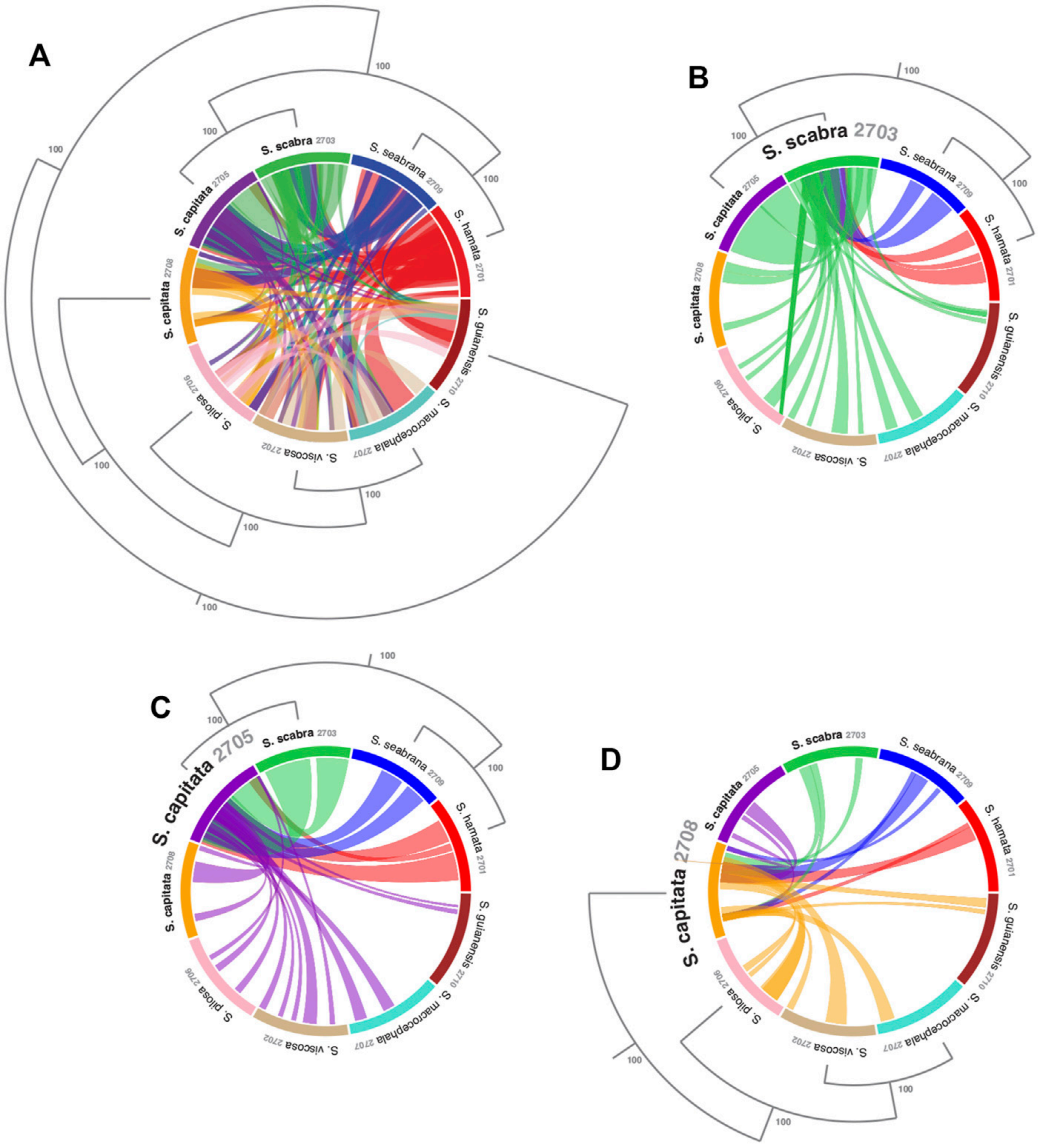
Visualizing sequence similarity among mitogenomes of *Stylosanthes* species with a focus on the relationships among the allotetraploids. **(A)** Mitogenomes synteny all-to-all. Synteny of *S*. *scabra* mitogenome **(B)**, *S*. *capitata* (SCA 2705) **(C)** and *S*. *capitata* (SCA 2708) **(D)** to the other species. Phylogenetic relationships were based on the topology of whole plastome alignment analyzed by Bayesian inference. Values in the nodes indicate posterior probabilities.

A circularized mitochondrial genome assembly was obtained for all nine samples and varied in length from 350,377 bp in *S*. *macrocephala* to 523,870 bp in *S*. *seabrana* (Supplementary Table S1). An extensive variation in the order of approximately 200 Kb was found among *Stylosanthes* mitogenomes with the following increasing order: *S*. *macrocephala* SMA 2707 (350,377 bp), *S*. *viscosa* SVI 2702 (353,136 bp), *S*. *capitata* SCA 2708 (384,410 bp), *S*. *pilosa* SPI 2706 (433,649 bp), *S*. *capitata* SCA 2705 (456,448 bp), *S*. *guianensis* SGU 2710 (468,896 bp), *S*. *scabra* SSC 2703 (492,899 bp), *S*. *hamata* SHA 2701 (503,967 bp) and *S*. *seabrana* SSE 2709 (523,870 bp) (Supplementary Table S1). Mitogenomes features of each studied species, including the number of transfer RNA (tRNA), ribosomal RNA (rRNA), and protein-coding genes from the annotated regions are shown in Figure 2 and Supplementary Table S2. Mitogenome maps for each sample are provided on Supplementary Figure S1.

To access mitochondrial genome structure variation in *Stylosanthes*, the syntenic relationship was analyzed within all nine *Stylosanthes* mitochondrial genomes using SyMAP (Figure 1). In general, low conservation of synthetic blocks (considered from the gene order) was observed in the analyzed *Stylosanthes* species (Figure 1A). We investigated comparatively the relationship of the mitogenomes of each allotetraploid with the other species of the genus (Figures 1B–D). Relatively high linearity was observed among the mitogenomes of the allotetraploids *S*. *scabra* SSC 2703 and *S*. *capitata* SCA 2705, and between them and the phylogenetically close diploids *S*. *hamata* SHA 2701 and *S*. *seabrana* SSE 2709 (Figures 1B,C). Surprisingly, no evidence of linearity between the mitogenome of the two *S*. *capitata* samples (SCA 2705 and SCA 2708) and its putative diploid progenitors *S*. *pilosa* SPI 2706 (E genome) or *S*. *macrocephala* SMA 2707 (D genome) was observed (Figures 1D).

### Chloroplast Genomes

**FIGURE 3:**
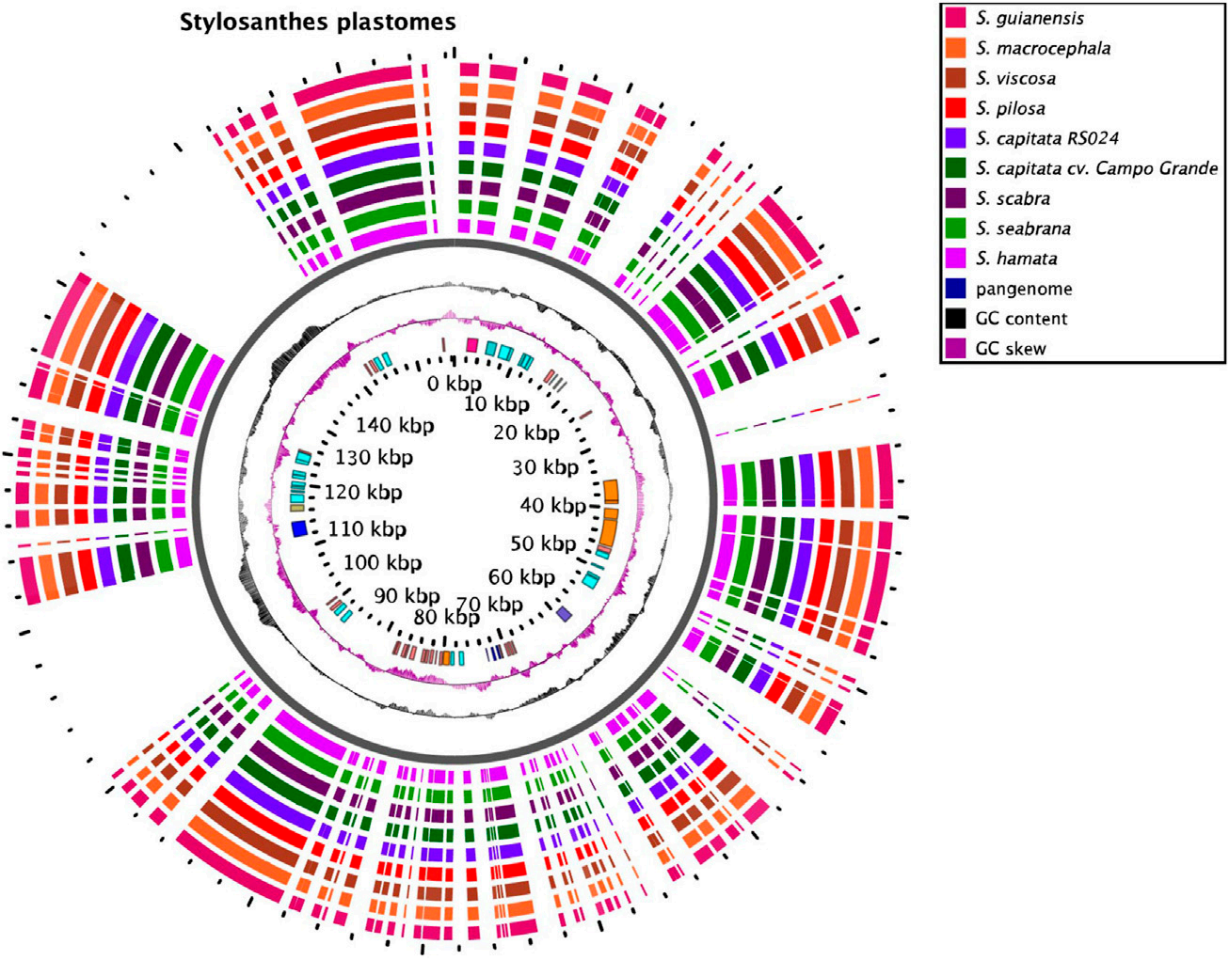
Plastome features of Stylosanthes. Annotated sequences are shown in the inner circle.

Plastomes of all species are very similar in length varying from 156,244 bp in *S*. *viscosa* to 156,502 bp in *S*. *hamata* and *S*. *scabra* (Figure 3; Supplementary Figure S2). No major macrostructural rearrangements were detected in the *Stylosanthes* genomes analyzed here. The potential of these plastomes for determining the (maternal) origin of allopolyploids has been explored from a phylogenetic point of view (see below).

### Genomic Repetitive Fraction Characterization and Comparative Analysis of Repeat Abundance

Individual clustering analysis with RepeatExplorer2 revealed that all nine *Stylosanthes* species shared a similar amount of repetitive sequences in their genomes (over 50%). In the present study, we have identified different families of repetitive elements, belonging to Class I (retroelements) and II (DNA transposons) mobile elements, as well as 5S and 35S rDNA and satDNA repeats (Table 2). The sample that showed the most repeat diversity was *S*. *macrocephala* (SMA 2707), with a total of 17 different classes of repeats, and the one with the lowest diversity was *S*. *guianensis* (SGU 2710), with only seven different classes (see Table 2).

**TABLE 2:** Genomic abundances (%) of the main repetitive sequences identified in the genomes of *Stylosanthes* and *A*. *hypogaea*. Bold values indicate the sum of each individual group of repeats as well as the total sum of repeat abundance.

Classes		Family	Genomic abundance (%)									
			S. hamata (SHA 2701)	S. viscosa (SVI 2702)	S. scabra (SSC 2703)	S. capitata (SCA 2705)	S. pilosa (SPI 2706)	S. macrocephala (SMA 2707)	*S*. *capitata* RS024 (SCA 2708)	*S*. *seabrana* (SSE 2709)	S. guianensis (SGU 2710)	*A*. *hypogaea*
LTR Ty1/Copia		SIRE	14,950	9,882	13,880	11,680	5,084	1,564	4,871	12,250	0.275	1,0.097
		Ikeros						0.022				0.356
		Bianca	1,208		2,418	1,938	1,133	0.449	1,679	0.750	0.027	0.541
		Ale		0.138	0.048	0.187	0.065	0.192	0.024			0.159
		TAR		0.042				0.138				0.022
		Ivana		0.015	0.157	0.222	0.021	0.115	0.049			
		Tork	0.204	0.756	0.495	0.757	0.575	1,100	0,0.398	0.276		0.185
			**Total**	**16,362**	**10,833**	**16,998**	**14,784**	**6,878**	**3,58**	**7,021**	**13,276**	**0,302**	**2,36**
LTR Ty3/Gypsy	non-chromovirus	Athila	39,450	31,890	31,730	31,780	24,750	27,220	34,670	37,940	35,220	46,170
		Ogre	0.294	0.106	0.310	0.728	0.397	0.177	0.028	0.088		
		Retand	0.286	1,904	0.401	1,303	1,959	3,021	1,573	0.433	1,424	2,929
	chromovirus	Tekay	0.151	4,999	0.738	4,002	9,799	6,794	5,919	0.194	0.920	3,565
		Galadriel	0.085		0.128	0.147		0.056	0.018	0.087		0.052
		CRM	0.014			0.118	0.091		0.092	0.037		
			**Total**	**40,28**	**38,899**	**33,307**	**38,078**	**36,996**	**37,268**	**42,3**	**38,779**	**37,564**	**52,716**
LTR non-classified		LTR		2,112								
		Ty1_copia							0.016			
Non-LTR		pararetrovirus		0.264		0.112	0.119		0.055			0.299
		LINE	0.033	0.311	0.422	0.730	0.107	0.367	0.378			0.369
**Total**			**56,675**	**52,419**	**50,727**	**53,704**	**44,1**	**41,215**	**49,77**	**52,055**	**37,866**	**55,744**
DNA transposons		MuDR_Mutator	0.255	1,400	0.333	0.811	1,148	1,393	0.909	0.155	0.744	0.379
		EnSpm_CACTA	0.206	0.618	0.119	0.555	0.548	0.375	0.276	0.419	1,182	
		PIF_Harbinger					0.030	0.025	0.016			0.014
		hAT						0.041				
		Helitron		0.030		0.025						0.039
DNA transposons non-classified		Classe_1	0.536	0.285	0.617	0.420	0.558	0.959	0.319	0.520	0.033	
**Total**			**0,997**	**2,333**	**1,069**	**1,811**	**2,284**	**2,793**	1,52	1,094	**1,959**	**0,432**
rDNA		45S_rDNA	0.426	0.666	0.312	0.706	0.981	3,551	1,070	1,236	0.658	0.805
		5S_rDNA	0.018	0.051	0.034	0.093	0.152	0.185	0.107	0.064	0.139	0.270
Satellite DNA			3,269	1,038	4,835	0.450	0.852	1,079	0.931	0.173	0.693	1,484
Non-classified			5,459	6,002	11,360	10,290	5,464	12,660	11,180	10,010	17,730	8,778
**Total of Repeats**			**66,844**	**62,509**	**68,337**	**67,054**	**53,833**	**61,483**	**64,578**	**64,632**	**59,045**	**67,513**

Athila (LTR – Ty3/gypsy) retroelements were by far the most abundant class of TEs found in all genomes, showing in all samples over 24% of genomic abundance (Table 2). Despite the high abundance of Athila found in *Stylosanthes* genomes, no clear relationship between diploids and allopolyploids was observed. The second most abundant class of repeat found in all genomes was the SIRE family, which belongs to the LTR–Ty1/copia clade (Table 2). In contrast to Athila, SIRE (LTR–Ty1/copia) abundance showed a stronger correlation between diploids and allopolyploids, where *S*. *scabra* complex showed a clear higher abundance of these repeats compared to *S*. *capitata* complex (Supplementary Figure S3). Moreover, the RE2 comparative analysis showed that indeed both total repeat and satellitome composition have similar content and abundance among phylogenetically related species (Supplementary Figure S4).

### Phylogenetic Relationships Based on Different Approaches

**FIGURE 4:**
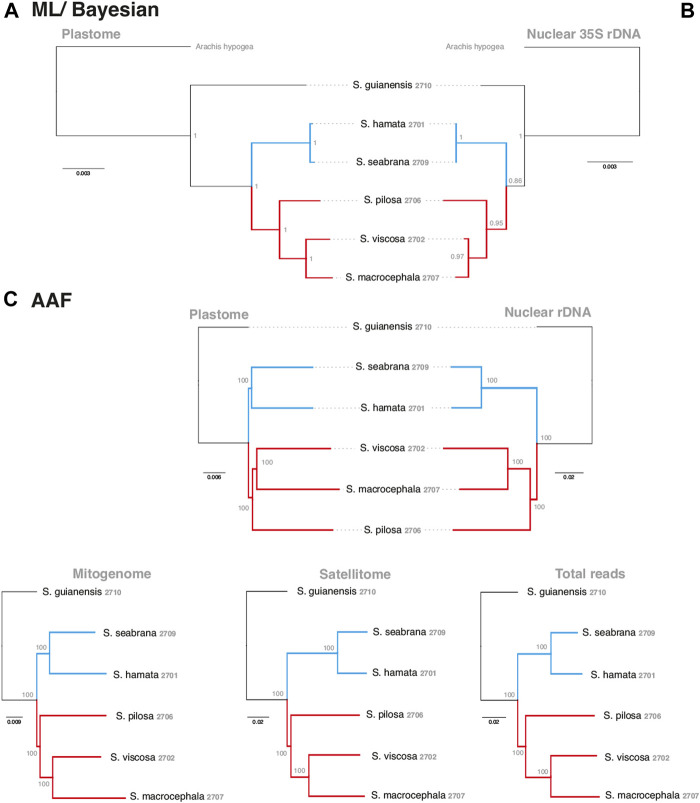
Alignment-based **(A**–**B)** and AAF **(C)** phylogenies of the *Stylosanthes* diploid species based on different datasets. Each dataset used in the respective inference is indicated on the top of each tree. Blue branches indicate A genome-specific and red branches group other genome types. Numbers indicate specific codes for each sample (see Table 1).

**FIGURE 5:**
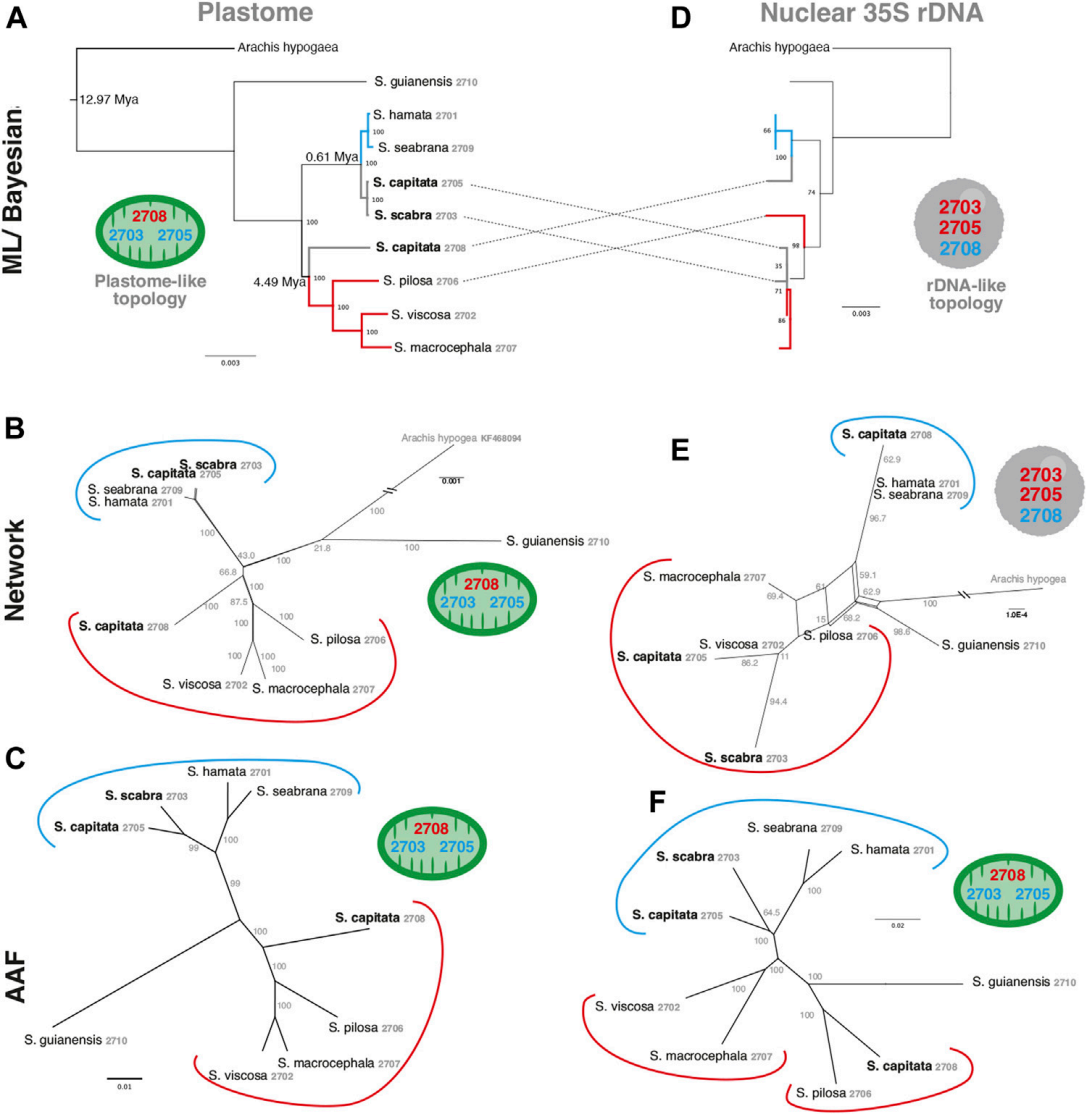
A comparison of alignment-based **(A**,**D)**, network analysis **(B**,**E)** and AAF **(C**,**F)** phylogenies of plastome **(left)** and rDNA **(right)** sequences including *Stylosanthes* allopolyploids. Ages of allopolyploid origins are indicated on Figure 5A. Blue branches indicate A genome-specific and red branches group other genome types. Numbers indicate specific codes for each sample (see Table 1). Green and gray circles indicate chloroplast-like or nuclear-like topologies, respectively.

In order to evaluate the use of alignment and assembly free analyses in the characterization of *Stylosanthes* allopolyploids, we have compared different approaches with more conventional alignment-based ones. Firstly, phylogenomic trees were constructed only for diploids species (*S*. *hamata* SHA 2701, *S*. *seabrana* SSE 2709, *S*. *pilosa* SPI 2706, *S*. *macrocephala* SMA 2707, and *S*. *viscosa* SVI 2702) based on alignment-dependent approaches. For Bayesian Inference (BI)/Maximum Likelihood (ML) we analyzed the following data sets: whole plastomes (Figure 4A) and nuclear rDNA sequences alignments (Figure 4B). To explore the AAF approach, that analyzes directly NGS data, we analyzed mapped reads from both plastomes and rDNA clusters as well as mitogenome, satellite and total reads (Figure 4C). All the diploid trees showed the same topology with high support showing *S*. *guianensis* SGU 2710 as the first diverging lineage of *Stylosanthes* and two main clades: *S*. *hamata* SHA 2701 + *S*. *seabrana* SSE 2709 (Figure 4 in blue) and *S*. *pilosa* SPI 2706 (*S*. *macrocephala* SMA 2707 + *S*. *viscosa* SVI 2702) (Figure 4 in red). Then, phylogenetic analyzes were performed including also the tetraploid samples (*S*. *capitata* SCA 2705, *S*. *capitata* SCA 2708, and *S*. *scabra* SSC 2703). In this case, we compared the plastidial and rDNA topologies using BI/ML, Splitstree4 network (considering potential reticulated evolution of rDNA), and AAF. We founded two main topologies. In the first one [named here as plastome-like] the allotetraploids *S*. *capitata* SCA 2705 and *S*. *scabra* SSC 2703 were positioned in the *S*. *hamata* + *S*. *seabrana* clade and the other sample of *S*. *capitata* (SCA 2708) was related to the *S*. *macrocephala* + *S*. *pilosa* + *S*. *viscosa* clade (Figures 5A–C). The second topology [named here as rDNA-like] oppositely positioned the allotetraploids: *S*. *capitata* SCA 2708 with *S*. *hamata* + *S*. *seabrana* and *S*. *capitata* SCA 2705 and *S*. *scabra* SSC 2703 with *S*. *macrocephala* + *S*. *pilosa* + *S*. *viscosa* clade (Figures 5D,E). Surprisingly, the topologies generated by AAF using different sets of reads (mitogenome, satellite, and total reads) were plastome-like (Figure 5F and Supplementary Figure S5). Because both *S*. *scabra* (SSC 2703) and *S*. *capitata* (SCA 2705) showed very similar genome structures, we were interested to learn if they had similar origin times. Therefore, we dated the plastome phylogeny (Figure 5A; Supplementary Figure S6). Indeed both *S*. *scabra* (SSC 2703) and *S*. *capitata* (SCA 2705) allopolyploids revealed a very recent origin time (0.61 Mya) compared to a more ancient origin for the *S*. *capitata* (SCA 2708), which revealed an origin time of at least 4.49 Mya.

### Satellite DNAs Characterization

**FIGURE 6:**
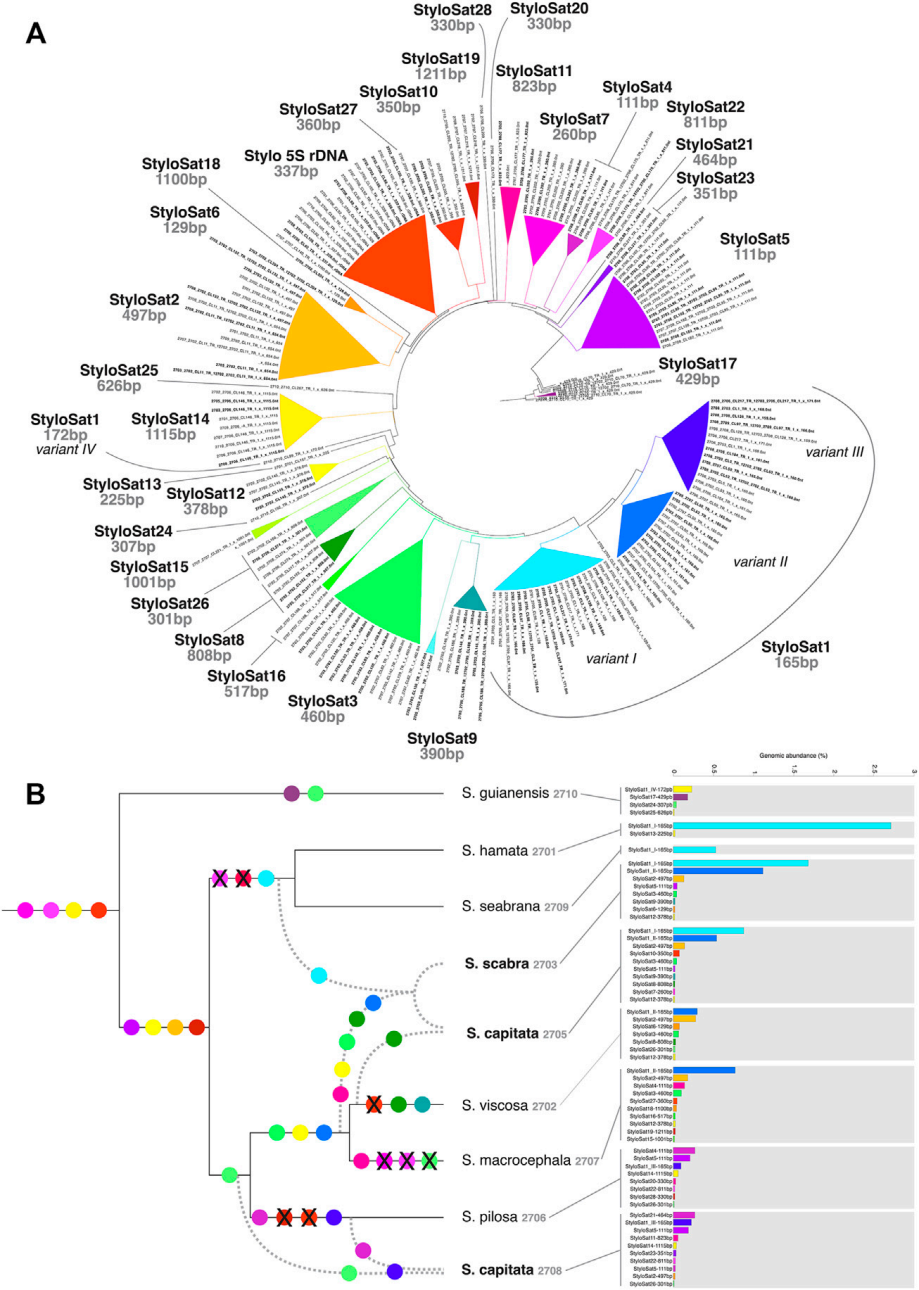
Satellitome characterization of *Stylosanthes*. **(A)** AAF tree showing the grouping of all different satDNA families found in *Stylosanthes*. StyloSat1 family is the most abundant and shows specific lineage similarity, which is conserved in allopolyploids. **(B)** Overview of satDNA showing more than 0.01% of genomic abundance in each sample identified by TAREAN. SatDNA consensus of each sample obtained by TAREAN/RepeatExplorer analysis were used as reference to retrieve repeat reads mapped uniquely to each consensus. The draw tree reflects the most parsimonious species relationships based on all our analyses.

The abundance of satDNA repeats varied in a species-specific manner from 0.17 to 4.8%, being the higher values observed in *S*. *hamata* and *S*. *scabra*, which showed 3.26% e 4.83%, respectively (Table 3). Despite the variance in genomic abundances of satDNA, there was no positive correlation between abundance and number of different satDNA familes found. For instance, *S*. *hamata* presented only two different satDNA repeats, with high genomic abundance, while *S*. *pilosa* for instance showed the highest number of different satDNA repeats, but relatively low genomic abundance (Table 3). Clearly, species of the clade I showed very few satDNA repeats (1–2) in *S*. *hamata* and *S*. *seabrana*, while species of the clade II showed higher numbers of different satDNA repeats (Figure 6).

**TABLE 3:** Comparison of genomic abundance (%) of satDNA repeats, by mapping all reads to consensus sequences identified by the TAREAN tool.

Species	SatDNA genomic abundance (%)	Number of satDNA repeats	Genome
*S*. *hamata* (SHA 2701)	2.90	2	A
*S*. *viscosa* (SVI 2702)	1.06	7	B
*S*. *scabra* (SSC 2703)	3.22	7	AB
*S*. *capitata* (SCA 2705)	1.91	7	AB
*S*. *pilosa* (SPI 2706)	0.86	8	E
*S*. *macrocephala* (SMA 2707)	1.64	8	D
*S*. *capitata* (SCA 2708)	1.00	7	DE
*S*. *seabrana* (SSE 2709)	0.66	1	A
*S*. *guianensis* (SGU 2710)	0.50	4	G

Figure 6 shows the satellitome diversity in *Stylosanthes* and the genomic abundance of each satDNA consensus repeat in each species. Based on the comparative analysis of the RepeatExplorer and AAF phylogeny we have identified a total of 28 satDNAs. For instance, a tandem repeat found only in species of the clade I (*S*. *hamata* and *S*. *seabrana*) is called “StyloSat1-variant I” with a monomer length of 165 bp. Thus, based on the grouping pattern of each satDNA we were able to identify possible synapomorphies in some clades and establish relationships between diploids and allopolyploids (Figure 6). Taking advantage of this approach, we could identify that the presence of StyloSat1-variant I in the allopolyploids *S*. *capitata* SCA 2705 and *S*. *scabra* SSC 2703 reveals the likely participation of species of clade I (*S*. *hamata* + *S*. *seabrana*) in the origin of these hybrids. On the other hand, the presence of the clade II specific satDNAs (e.g. StyloSat1-variant II, StyloSat2-497bp, StyloSat3-460bp, and StyloSat12-378bp) in these allopolyploids indicates the additional contribution of species from clade II to the origin of these hybrids. The allotetraploid *S*. *capitata* SCA 2708 showed a satellitome composition more similar to species from clade II, specifically to *S*. *pilosa* SPI 2706, to which it shares the satDNAs StyloSat1-variant III, StyloSat5-111bp e StyloSat14-1115bp among others.

**FIGURE 7:**
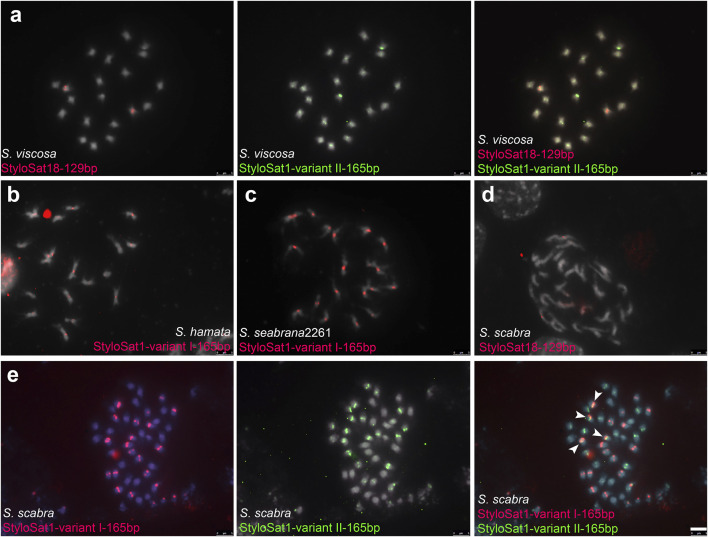
FISH in Stylosanthes scabra complex. **(A)** FISH in *S*. *viscosa* with *S*. *viscosa*-specific satDNA repeats StyloSat18-129bp and StyloSat1-variant II-165bp. FISH in *S*. *hamata* **(B)** and *S*. *seabrana* **(C)** with *S*. *hamata/seabrana*-specific satDNA repeat StyloSat1-variant I-165bp. FISH in *S*. *scabra* with StyloSat18-129bp **(D)** and with StyloSat1-variant I-165bp and StyloSat1-variant II-165bp **(E)** satDNA repeats. Bar = 5 μm.

To test whether the differential accumulation of satDNA repeats observed in *Stylosanthes* allopolyploids correspond to their origin, we selected three satDNA repeats exclusively found in *S*. *viscosa* (StyloSat1-variant II-165bp and StyloSat18-129bp), *S*. *hamata*, and *S*. *seabrana* (StyloSat1-varian I-165bp) and test for their presence and distribution in *S*. *scabra* 2703. Based on our above analysis of satDNA sequences, we know that StyloSat1-variant I-165bp and StyloSat1-variant II-165bp actually belongs to the same family of satDNA sharing 70% of sequence similarity after alignment of their consensus sequences (Figure 6; Supplementary Figure S7). After FISH hybridization StyloSat18-129bp showed proximal signals in a single pair of *S*. *viscosa* chromosomes while StyloSat1-variant II-165bp was seen at the centromeric region of all chromosome pairs (Figure 7A). StyloSat1-varian I-165bp showed signals in centromeric regions of all *S*. *hamata* (Figure 7B) and *S*. *seabrana* (Figure 7C) chromosomes, reinforcing the genomic similarity of these two species. In *S*. *scabra* we could also confirm the presence of a single chromosome pair showing centromeric signals for StyloSat18-129bp (Figure 7D). Remarkably, StyloSat1-variant I-165bp and StyloSat1-variant II-165bp satDNA repeats were also seen at centromeric regions of *S*. *scabra* chromosomes, where most chromosomes showed either one of the repeats (Figure 7E). However, two chromosome pairs showed both repeats sitting at their centromeric regions (Figure 6E, arrowheads). Although in these two pairs the signals are found on their (peri)centromeric regions they do not overlap completely, suggesting that they are indeed present in the same chromosome and it is not a cross-hybridization artefact. Also, if a cross-hybridization had occurred, we should expect it for most chromosomes and not for these two pairs specifically.

## Discussion

### The Use of Repeat-Based AAF to Understand Phylogenetic Relationships in Stylosanthes: Clarity in Diploids Versus Uncertainty in Allopolyploids

Our data show a strong congruence in the phylogenetic relationships of *Stylosanthes* diploid species, regardless of the phylogenetic reconstruction method (BI, ML, and AAF) or dataset (rDNA, plastome, mitogenome, satellitome, or total reads). This suggests that, at the diploid level, the reconstruction of fully bifurcate topology results from an expected hierarchical process of speciation. The diploid clades are morphologically supported. *Stylosanthes guianensis* differs from the other species by the fruit having only one fertile article, a scanty rostrum, and the epidermis of the fruit covered with papillae. The other present two fertile articles, the lower article pubescent, the upper article without papillae and developed rostrum. *S*. *hamata* and *S*. *seabrana* share many characters as the presence of a rudimentary axis, 2 inner bracteoles, ellipsoid spikes, uncinate rostrum, viscid bristles on the stems and erect habit. They differ mainly by the rostrum exceeding the loment length in *S*. *hamata*, while in *S*. *seabrana*, the rostrum is shorter (Maass and Mannetje, 2002; Costa, 2006). *Stylosanthes macrocephala*, *S*. *viscosa*, and *S*. *pilosa* share fruits with prominent reticulation (Costa, 2006). On the other hand, *S*. *macrocephala* and *S*. *pilosa* presents the rudimentary axis, while it’s absent in *S*. *viscosa*. *Stylosanthes viscosa* and *S*. *pilosa* present coiled rostrum, while it is uncinate in *S*. *macrocephala*. These results support other phylogenetic studies that indicate that the presence of the axis is not a valuable character to determine infrageneric classifications as it was previously used in taxonomic revisions (Mohlenbrock, 1958; Vander Stappen et al., 1999).

The inclusion of allopolyploids results in low support values, phylogenetic uncertainty, and incongruence between nuclear and organellar topologies, as reported in other studies (Marques et al., 2018; Souza et al., 2019). Establishing synapomorphies for these clades including the allopolyploids is very challenging regarding the different topologies between plastid and nuclear markers. Furthermore, the non-monophyletism of *S*. *capitata* brings difficulties to recognize morphological traits unique to the clades. However, analyzing the plastome phylogeny (Figure 5), we can observe that the clade *S*. *hamata* + *S*. *seabrana* + *S*. *scabra* share exclusive features like uncinate rostrum in the fruit, the upper article densely pubescent, with long non-glandular trichomes, and the outer bracteole trifid. The clade *S*. *capitata* + *S*. *macrocephala* + *S*. *viscosa* + *S*. *pilosa* can be supported by the bifid outer bracteole and persistent leaflet at the bracts. The bracts of *S*. *macrocephala* and *S*. *capitata* are wider than longer, meanwhile the bracts of *S*. *pilosa* and *S*. *viscosa* don’t reach more than 10 mm. Perhaps the inclusion of more species in the phylogeny may shed light to the synapomorphies of these clades. Nevertheless, the plastome topology better represents the morphology than the nuclear tree.

A series of phylogenetic studies using the Sanger sequencing approach (based on nuclear ITS or plastid loci) has failed to achieve well-resolved topologies for the genus *Stylosanthes* (Liu et al., 1999; Vander Stappen et al., 1999; Vander Stappen et al., 2002), which is probably related to the inclusion of these allopolyploids in the analyzes and low diversification of the sequences and markers used. Approximately 15% of speciation events in angiosperms have been estimated to be associated with polyploidy (Wood et al., 2009) and allopolyploidy has long been considered to be one of the most frequent events (Barker et al., 2016; Doyle and Sherman-Broyles, 2017; Oxelman et al., 2017). Failure to account for allopolyploidy when reconstructing the past evolution of groups where it has occurred inevitably will lead to inaccurate phylogenetic hypotheses. For cases of uncertain phylogenetic relationships, as in *Stylosanthes*, network analysis has been proposed, considering that the algorithms in this type of analysis assume reticulated evolution (Oxelman et al., 2017; Souza et al., 2019).

Alignment-based phylogenies are still the main method to reconstruct phylogenetic relationships among species (Delsuc et al., 2005; Yang and Rannala, 2012), whereas they are a limited approach in allopolyploid-rich groups. Although well-supported species relationships can be resolved with such approach, a clear limitation when aligning huge numbers or highly divergent sequences, like repeats, is still a major barrier. Thus, most alignment-based phylogenies are based on single genes or more robust ones based on target capture datasets or whole organelle alignments (Saarela et al., 2018; Johnson et al., 2019; Liu et al., 2019). More recently, alternative methods based on alignment and assembly free phylogenetic reconstructions have emerged to deal with this problem (Fan et al., 2015; Lau et al., 2019; Sarmashghi et al., 2019). However, to date, only a single tool is able to construct phylogenetic trees as well as report supporting values for these trees, the AAF algorithm (Fan et al., 2015). Remarkably, the use of alignment and assembly free phylogenies has not yet been explored specifically for repeat sequences. Repeat-based phylogenies have recently become the focus of several studies and are quite accurate in reporting the right phylogenetic relationship among species. The application of those methods is normally based on either repeat abundance (Dodsworth et al., 2015; McCann et al., 2020) or repeat similarity (Vitales et al., 2020). Here, we have shown that AAF-based phylogenomics can be applied as a powerful tool to analyze both sets of WGS reads as well as repeat- and organelle-derived reads. All our AAF phylogenies revealed strongly supported clades, mostly in agreement with alignment-based phylogenies. Furthermore, we propose that the AAF approach could be nicely combined with the output of different repeat identification tools, like RepeatExplorer, where isolated reads from specific repeat lineages can be combined to generate repeat-based phylogenies (e.g., satDNA, LTR retrotransposons, total repeats, etc.). AAF phylogenies can also be applied in order to identify satDNA families and overall satDNA diversification in specific groups as well as other repeat families, as shown here.

The existence of incongruent nuclear (rDNA-like) and organellar (plastome-like) phylogenetic topologies was already demonstrated for *Stylosanthes* (Marques et al., 2018). However, it was remarkable that different repeat sets (mitogenome, satellitome, total reads, and total repeats) revealed similar plastome-like topologies [except 5S rDNA reads that generated a unique topology (Supplementary Figure S5)]. Assuming that this topology reflects a maternal genealogy, we found evidence of maternal bias in the repeat abundances. As part of the “genomic shock” experienced by combining two divergent subgenomes within one neoallopolyploid nucleus, there are predictions regarding the level of genome reorganization, and sequence loss/retention based on the direction of the cross (Dodsworth et al., 2020). The maternal subgenome is expected to be favored, relative to the paternal subgenome, due to its compatibility within the maternal cytoplasm, as suggested by Nuclear Cytoplasmatic Interaction (NCI) hypothesis (Lim et al., 2004). This potentially leads to specific degradation of various elements from the paternal subgenome, as predicted by this NCI hypothesis (Lim et al., 2004; Renny-Byfield et al., 2011; Renny-Byfield et al., 2012).

Apparently, the allopolyploid age can influence the restructuring of the genome and turnover of repetitive elements. In older tetraploids, repeat dynamics are much more variable, impacting for example on genome size. However, in young allopolyploids repeat abundances are close to the sum of abundances expected from both parental donors (Dodsworth et al., 2020). Thus, the uncertainty in identifying the parents of an allopolyploid is proportional to the age of formation of that hybrid (McCann et al., 2018). We demonstrate here that the origin of *S*. *scabra* 2703 + *S*. *capitata* 2705 is more recent (0.61–1.71 Mya) than *S*. *capitata* 2708 (3.01–4.49 Mya), which may explain the greater complexity in detecting the parents of this last allotetraploid.

### SatDNA Evolution in Stylosanthes

Satellite DNA has been used as an important source for subgenome identification in allopolyploids and as phylogenetic information (Gill et al., 2009; Koukalova et al., 2010). Here we found that satellite DNA showed a great diversification in the genus *Stylosanthes* and only a single family (StyloSat1) was found in all samples analyzed and seems to be conserved among the species, most likely representing the centromeric DNA. Indeed, StyloSat1 demonstrated a subgenome-specific sequence conservation and allowed us to detect the A and B subgenomes of *S*. *scabra*. Also, *S*. *hamata* and *S*. *seabrana* showed similar hybridization profiles with StyloSat1, although the *S*. *hamata* specific satDNA StyloSat13, which was observed in a single chromosome pair, was not identified in *S*. *seabrana*. The high abundance, phylogenetic conservation and physical location in the proximal region of the chromosomes suggests that this satellite is the centromeric DNA of these species.

Most of the other satDNA families were shared only among species with a high degree of relationship, in this case between hybrids and their genome donors. Cases of absence of satellite DNAs in certain genomes inconsistent with phylogeny (see Figure 6) can be explained by the library hypothesis (Fry and Salser, 1977). Remarkably, satDNA sequences showed a high phylogenetic signal in our AAF analyses and helped to identify diploid progenitors. Similarly, a recent study also reported a high phylogenetic signal for satDNA abundance among *Melampodium* L. (Asteraceae) species (McCann et al., 2020). Our AAF approach seems highly relevant and robust for repeat-based phylogeny since it takes into account both similarity and abundance as well as reporting a support value for the phylogenetic tree.

### The Macrostructure of Mitochondrial Genomes and Genomes Reflect Completely Different Evolutionary Histories in Stylosanthes

Here we make available for the first time the complete mitogenomes for nine species and further provides additional six plastomes for *Stylosanthes* species, ensuring fundamental information for the next studies of systematics and genetic breeding of the genus. Mitochondrial genomes of the studied species of *Stylosanthes* have all similar genic contents despite a low density of genic sequences like most plants. Variations in genome sizes accounted for the most diversity found among the characterized *Stylosanthes* mitogenomes. These large variations are common; however, they do not directly indicate the number of functional genes, yet they are more related to intergenic regions. Indeed, the mitochondrial genome of higher plants shows extreme variations in its structure, size, and complexity (Gualberto and Newton, 2017). Specific features of mitochondrial genomes such as their high rates of recombination can account for several aspects of plant mitochondrial genome structure. Thus, playing a major role in the evolution of plant mitochondrial genomes (Arrieta-Montiel and Mackenzie, 2011; Knoop et al., 2011; Kuhn and Gualberto, 2012). Based on our synteny maps for mitogenomes, a similar situation to plastomes was observed, with the samples *S*. *hamata*, *S*. *seabrana* showing the highest synteny, confirming their close relationship. Also, *S*. *scabra* and *S*. *capitata* (SCA 2705) shared high synteny to both A genome diploid mitogenomes, confirming their maternal A genome origin. Therefore, we reinforce the use of organelle genomes for evolutionary and phylogenetic studies in this group.

The *Stylosanthes* chloroplast genomes were very conserved in genetic content, total length, and organization, as is found for other plant groups as well (Daniell et al., 2016). We confirmed our previous findings that a diploid species with A genome (*S*. *hamata* or *S*. *seabrana*) is likely the maternal genomic donor of *S*. *scabra*, as their plastomes shared 99.798 and 99.776% pairwise identity, respectively. Additionally, the *S*. *capitata* (SCA 2705) most likely has the same maternal progenitor as *S*. *scabra* due to a higher similarity of its organelle genetic information with A genome species *S*. *hamata* and *S*. *seabrana*, 99.803 and 99.783%, respectively. In contrast, it showed less pairwise identity with *S*. *pilosa* and *S*. *macrocephala*, 98.161%, and 98.242%, respectively. Although *S. scabra* and *S. capitata* (SCA 2705) shared the highest pairwise identity in their plastomes, the divergence in their mitogenomes suggests that these allopolyploids have independent origins. Furthermore, the *S*. *capitata* (SCA 2708), which was previously indicated to have DDEE genome composition (Liu and Musial, 2001) did not show the same level of sequence similarity to its putative genome donors *S*. *macrocephala* (98.317%, D genome) or *S*. *pilosa* (98.334%, E genome), but it grouped in the same clade with both and *S*. *viscosa* in the organelle genome phylogenies.

### Genomic Repetitive Fraction Characterization and Comparative Analysis of Repeat Abundance

All nine samples of the genus *Stylosanthes* Sw. characterized in this study showed a high abundance of repetitive DNA and maintain the conservation of many sequences. Ty3/gypsy Athila elements were the most abundant in all species. Ty1/copia SIRE elements were highly abundant in species with A genome composition, clearly being a shared feature of A genome species, while in the other genomes this element showed variable lower abundances. Among all genomes, *S*. *guianensis* showed the highest divergence within the genus and agrees with its more distant relationship with the other species. In general, the overall repeat abundance matched the species relationships, while polyploids tend to accumulate repeats from both diploid progenitors.

In general, species with A genome had more abundance of total satDNA but less satDNA diversity compared to the species with other genome types. SatDNA abundances varied greatly between the two very closely related *S*. *hamata* and *S*. *seabrana*. Although the major family of *Stylosanthes* satDNA (StyloSAT1) was found in both species, its abundance varied from 3% in *S*. *hamata* to 0.7% in *S*. *seabrana*, suggesting that an amplification or deamplification has occurred since the separation of these two species. However, our FISH analysis confirmed the presence of this satDNA at the centromeric regions of all chromosomes in both species.

### Taxonomic Conflicts

Concerning the samples that compose the cv. Campo Grande, *S*. *capitata* (SCA 2705) and *S*. *macrocephala* (SMA 2707), which has been marketed by Embrapa Beef Cattle since 2000 (EMBRAPA, 2000), we suggest that a taxonomic review should be carried out since our studies show some inconsistencies with these taxonomical designations. *S*. *capitata* is morphologically similar to *S*. *macrocephala* and *S*. *pilosa* by the craspedodromous leaflets, suborbicular bracts, wider than longer, two long, fertile loments, and uncinate rostrum. *Stylosanthes macrocephala* and *S*. *capitata* were even considered synonymous in some studies (Vanni, 2017). Our results corroborate the distinction of the two species, but brings attention to the proximity of *S*. *capitata* (SCA 2708) and *S*. *pilosa*. Both species are very alike, sometimes hard to differentiate. The main characteristic to distinguish them is the indument that is pilose in *S*. *pilosa* with long bristles and trichomes (Ferreira and Costa, 1977). Further taxonomic studies may elucidate the difference between them. In our study, we found that *S*. *capitata* (SCA 2705) is more genetically similar to *S*. *scabra*. The two species are distinguishable by bract width (wider in *S*. *capitata*, with more than 10 veins) and stem indument (scabrous with short bristles in *S*. *scabra*). Therefore, a taxonomic review is necessary so that consumers are sure of the product they are purchasing, and also so that the company can adapt the recommendations for that particular cultivar, according to the species that actually compose it.

Despite the divergence between some of the results we obtained and those in the literature, as in the case of *S*. *seabrana*, it cannot be said whether it is a distinct species or a synonym of *S*. *hamata* in the process of speciation. Taking all our findings into consideration we confirm the complexity and difficulty of studies in the genus *Stylosanthes* and reinforces the need for more in-depth studies that review the taxonomy and phylogenetics of the group, and phylogenetics of the group in the light of genomic data.

## Data Availability

The datasets presented in this study can be found in online repositories. The names of the repository/repositories and accession number(s) can be found in the article/Supplementary Material.
