# Supplementary material for: High-Throughput Genomic Data Reveal Complex Phylogenetic Relationships in Stylosanthes Sw (Leguminosae)
Source: Front Genet. 2021 Sep 23;12:727314. doi: 10.3389/fgene.2021.727314 (PMC8495327; doi:10.3389/fgene.2021.727314)
Supplement: Supplementary file 1 [file DataSheet2.PDF]

Suppl. Table 1. Mitochondrial genome features of *Stylosanthes*

	S. hamata	*S. viscosa*	*S. pilosa*	*S. capitata*	*S. scabra*	*S. macrocephala*	*S. capitata* RS024	*S. guianensis*	*S. seabrana*
Total size (bp)	503,967	353,136	433,649	456,448	492,899	350,377	345,666	468,896	523,870
Size of coding regions (bp)	39,000	35,478	35,772	35,130	38,484	32,690	21,760	35,892	37,896
Size of protein-coding regions (bp)	43,500	42,643	37,355	34,338	40,699	36,565	34,630	44,041	43,821
Size of rRNA (bp)	5,042	5,042	5,038	2,945	5,042	5,042	5,047	5,033	5,042
Size of tRNA (bp)	1,336	1,347	1,665	1,601	1,422	1,275	1,150	1,561	1,413
Size of intergenic regions (bp)	416,179	271,902	357,050	382,107	406,738	276,311	269,804	380,816	435,298
No. of different genes	33	33	33	31	33	30	32	32	32
No. of different protein-coding genes	47	46	47	42	47	45	47	47	47
No. of different tRNA genes	16	16	16	15	15	15	15	16	16
No. of different rRNA genes	3	3	3	1	3	3	3	3	3

No. of different duplicated genes	5	3	4	4	5	3	1	5	5
No. of different genes with introns	6	6	6	5	6	5	7	6	6
Overall % GC content	44.9	45.1	45.2	45.0	45.1	45.2	45.3	45.0	44.9
% GC content in protein-coding regions	46.4	46.4	45.7	45.8	46.5	45.3	46.0	46.3	46.4
% GC content in intergenic regions	46.5	46.5	46.1	46.2	46.5	45.9	45.4	46.6	46.6
% GC content in rRNA	52.1	52.1	52.0	51.1	52.1	52.1	44.0	52.1	52.1
% GC content in tRNA	51.5	52.2	49.1	49.7	52.4	52.2	49.5	52.1	52.1

Suppl. Table 2. Genes of *Stylosanthes* mitochondrial genomes.

GENE GROUP	GENE NAME								
	S. hamata	*S. viscosa*	*S. scabra*	*S.capitata* CG	*S. pilosa*	*S. macrocephala*	*S. capitata 24*	*S. seabrana*	*S. guianensis*
**Transfer RNAs**	trnA-UGC, trnD-cp, trnE, trnF, trnfM (2x), trnG, trnH, trnl, trnK (2x), trnM-cp, trnN-cp, trnP, trnQ, trnS, trnW-cp, trnY.	trnD-cp, trnE, trnF, trnfM, trnG, trnH, trnl, trnK (2x), trnM-cp, trnN-cp, trnP (2x), trnQ, trnS, trnW-cp, trnY.	trnD-cp, trnE, trnF, trnfM (3x), trnG, trnH, trnl, trnK (2x), trnM-cp, trnN-cp, trnP (2x), trnQ, trnS, trnW-cp, trnY.	trnD-cp, trnE, trnF, trnfM (2x), trnG, trnH, trnI, trnK (2x), trnM-cp, trnN-cp, trnP (2x), trnQ, trnS, trnY	trnA, trnD-cp, trnE, trnF, trnfM, trnG, trnH, trnI, trnK, trnM-cp , trnN-cp, trnP (2x), trnQ, trnS, trnW-cp (2x), trnY	trnD-cp, trnE, trnF, trnfM (2x), trnG, trnH, trnI, trnK, trnM-cp, trnN-cp, trnP (2x), trnQ, trnS, trnW-cp, trnY	trnD-cp, trnE, trnF, trnfM (2x), trnG, trnH, trnl, trnK , trnM-cp, trnN-cp, trnP, trnQ, trnS, trnW-cp, trnY.	trnD-cp, trnE, trnF, trnfM (2x), trnG, trnH-GUG, trnl, trnK (2x), trnM-cp, trnN-GUU, trnP (2x), trnQ-UUG, trnS, trnY, trnY-GUA.	trnD-GUC, trnE-UUC, trnF-GAA, trnF-GAA, trnfM (2x), trnG-GCC, trnH-GUG, trnl, trnK-UUU (2x), trnM-CAU, trnN-GUU, trnP (3x), trnP-UGG, trnQ-UUG, trnS-GCU, trnW-CCA, trnY-GUA.
**RNA polymerase**	rrn5, rrn18, rrn26.	rrn5, rrn18, rrn26.	rrn5, rrn18, rrn26.	rrn26	rrn5, rrn18, rrn26	rrn5, rrn18, rrn26	rrn5, rrn18, rrn26.	rrn26.	rrn5S, rrn18, rrn26.
**Ribossomal Protein small subunit**	rps1, rps3 (2x), rps4, rps10, rps12 (2x), rps14.	rps1, rps3 (2x), rps4, rps10, rps12.	rps1, rps3, rps4, rps10, rps12, rps14.	rps1, rps3, rps4, rps10, rps12, rps14	rps1, rps3 (2x), rps4, rps12, rps14	rps1, rps3 (2x), rps4, rps10, rps12	rps1, rps3, rps4, rps10, rps12, rps19.	rps1, rps3 (2x), rps4, rps10, rps12 (3x), rps14.	rps1, rps3 (2x), rps4, rps10, rps12, rps14.
**Ribossomal Protein large subunit**	rpl5, rpl16.	rpl5, rpl16.	rpl5, rpl16.	rpl5, rpl16	rpl5, rpl16	rpl5, rpl16	rpl5, rpl16.	rpl5, rpl16.	rpl5, rpl16.
**ATP synthase**	atp4, atp6, atp8, atp9, atpA.	Atp4, atp6, atp8, atp9, atpA.	Atp4, atp6, atp8, atp9, atpA.	Atp4, atp6, atp8, atp9.	Atp4, atp6, atp8, atp9, atpA	Atp4, atp6, atp8, atp9, atpA	atp4, atp6, atp8, atp9, atpA.	Atp4, atp6, atp8, atp9, atpA.	Atp4, atp6, atp8, atp9, atpA.
**Maturase**	matR	matR	matR	matR	matR	matR	matR	matR	matR
**Complex I (NADH dehydrogenase)**	nad3, nad4L, nad6, nad7, nad9	nad3, nad4L, nad6, nad7, nad9	nad3, nad4L, nad6, nad7, nad9	nad3, nad4L, nad6, nad7, nad9	nad3, nad4L, nad6, nad7, nad9	nad3, nad4L, nad6, nad7, nad9	nad3, nad4L, nad6, nad7, nad9	nad3, nad4L, nad6, nad7, nad9	nad3, nad4, nad6, nad7, nad9
**Complex II**	sdh4	sdh4	sdh4 (2x)	sdh4	sdh4	sdh4	sdh4	sdh4	sdh4

**Complex III (Ubichinol cytochrome c redutase)**	cob	cob	cob	_	cob	cob	cob	-	cob
**Complex IV (cytochrome c oxidase)**	cox1, cox2, cox3.	cox1, cox2, cox3.	cox1, cox2, cox3 (2x).	cox1, cox2, cox3.	cox1, cox2 (2x), cox3	cox1, cox2, cox3	cox1, cox2, cox3.	cox1, cox2, cox3.	cox1, cox2 (2x), cox3.
**Other genes**	ccmB, ccmC, ccmFc, ccmFn, tatC.	ccmB, ccmC, ccmFc, ccmFn, tatC.	ccmB, ccmC, ccmFc, ccmFn, tatC.	ccmB, ccmC, ccmFc, ccmFn, tatC.	ccmB, ccmC, ccmFc, ccmFn, tatC.	ccmB, ccmC, ccmFn, tatC.	ccmB, ccmC, ccmFc, ccmFn, tatC.	ccmB, ccmC, ccmFc, ccmFn, tatC.	ccmB, ccmC, ccmFc, ccmFn, tatC.

Suppl. Table 3. Plastome features of *Stylosanthes*.

	S. hamata	*S. viscosa*	*S. scabra*	*S. capitata* CG	*S. pilosa*	*S. macrocephala*	*S. capitata* RS024	*S. seabrana*	*S.guianensis*
Total size in bp	156,502	156,244	156,502	156,490	156,058	156,451	156,636	156,499	156,785
LSC size in bp	86,047	85,912	86,064	86,054	85,732	86,090	85,448	85,973	85,529
SSC size in bp	18,749	18,710	18,734	18,733	18,713	18,719	18,756	18,739	18,758
IR length in bp	25,853	25,811	25,852	25,851	25,806	25,821	25,829	25,853	25,874
Size of coding regions in bp	75,984	75,897	75,921	75,009	74,964	75,036	75,981	70,500	76,544
Size of protein-coding regions in bp	96,683	96,704	96,758	98,121	97,449	98,171	96,759	91,318	95,082
Size of rRNA in bp	9,062	9,062	9,062	9,062	9,062	9,062	9,062	9,062	9,062
Size of tRNA in bp	2,735	2,737	2,735	2,737	2,735	2,735	2,735	2,735	2,735
Size in bp of intergenic regions	22,693	22,313	22,171	22,483	22,500	23,493	31,397	28,097	19,179
No. of different genes	105	113	107	111	112	105	106	105	106
No. of different protein-coding genes	76	79	76	73	73	72	75	75	76
No. of different tRNA genes	29	31	29	29	30	29	22	30	31
No. of different rRNA genes	4	4	4	4	4	4	4	4	4
No. of different genes	12	12	12	12	12	12	12	12	12

duplicated by IR									
No. of different genes with introns	8	8	8	8	7	7	7	8	7
Overall % GC content	36,6	36,6	36,6	36,6	36,6	36,6	36,5	36,6	36,4
% GC content in protein-coding regions	37,8	37,8	37,8	37,8	37,8	37,8	37,8	37,8	37,8
% GC content in IGSs	38,5	38,5	38,5	38,5	38,6	38,6	38,6	38,6	38,1
% GC content in rRNA	55,5	55,5	55,5	55,5	55,5	55,5	55,5	55,5	49,5
% GC content in tRNA	53,2	53,2	53,2	53,2	53,2	53,2	53,2	53,2	39,4

Suppl. Table 4. Genes of *Stylosanthes* chloroplast genomes.

**Gene group**		***S. hamata***	***S. viscosa***	***S. scabra***	***S. capitata* GC**	***S. pilosa***	***S. macrocephala***	***S. capitata* RS024**	***S. seabrana***	***S. guianensis***
**Protein synthesis and DNA replication**	Transfer RNAs	trnA-UGC (2x), trnC-GCA, trnD-GUC, trnE-UUC, trnF-GAA, trnfM-CAU, trnG-UCC, trnH-GUG, trnI-CAU (2x), trnI-GAU (2x), trnK-UUU, trnL-CAA (2x), trnL-UAA, trnL-UAG, trnM-CAU, trnN-GUU (2x), trnP-UGG, trnQ-UUG, trnR-ACG (2x), trnR-UCU, trnS-GCU, trnS-GGA, trnS-UGA, trnT-GGU, trnT-UGU, trnV-GAC (2x), trnV-UAC, trnW-CCA, trnY-GUA	trnA-UGC (2x), trnC-GCA, trnD-GUC, trnE-UUC, trnF-GAA, trnfM-CAU, trnG-UCC, trnH-GUG, trnI-CAU (2x), trnI-GAU (2x), trnK-UUU, trnL-CAA (2x), trnL-UAA, trnL-UAG, trnM-CAU, trnN-GUU (2x), trnP-UGG, trnQ-UUG, trnR-ACG (2x), trnR-UCU, trnS-GCU, trnS-GGA, trnS-UGA, trnT-GGU, trnT-UGU, trnV-GAC (2x), trnV-UAC, trnW-CCA, trnY-GUA	trnA-UGC (2x), trnC-GCA, trnD-GUC, trnE-UUC, trnF-GAA, trnfM-CAU, trnG-UCC, trnH-GUG, trnI-CAU (2x), trnI-GAU (2x), trnK-UUU, trnL-CAA (2x), trnL-UAA, trnL-UAG, trnM-CAU, trnN-GUU (2x), trnP-UGG, trnQ-UUG, trnR-ACG (2x), trnR-UCU, trnS-GCU, trnS-GGA, trnS-UGA, trnT-GGU, trnT-UGU, trnV-GAC (2x), trnV-UAC, trnW-CCA, trnY-GUA	trnA-UGC (2x), trnC-GCA, trnD-GUC, trnE-UUC, trnF-GAA, trnfM-CAU, trnG-UCC, trnH-GUG, trnI-CAU (2x), trnI-GAU(2x), trnK-UUU, trnL-CAA (2x), trnL-UAA, trnL-UAG, trnM-CAU, trnN-GUU (2x), trnP-UGG, trnQ-UUG, trnR-ACG (2x), trnR-UCU, trnS-GCU, trnS-GGA, trnS-UGA, trnT-GGU, trnT-UGU, trnV-GAC (2x), trnV-UAC, trnW-CCA, trnY-GUA	trnA-UGC (2x), trnC-GCA, trnD-GUC, trnE-UUC, trnF-GAA, trnfM-CAU, trnG-UCC, trnH-GUG, trnI-CAU (2x), trnI-GAU (2x), trnK-UUU, trnL-CAA (2x), trnL-UAG, trnM-CAU, trnN-GUU (2x), trnP-UGG, trnQ-UUG, trnR-ACG, trnR-ACG, trnR-UCU, trnS-GCU, trnS-GGA, trnS-UGA, trnT-GGU, trnT-UGU, trnV-GAC (2x), trnV-UAC, trnW-CCA, trnY-GUA	trnA-UGC (2x), trnC-GCA, trnD-GUC, trnE-UUC, trnF-GAA, trnfM-CAU, trnG-UCC, trnH-GUG, trnI-CAU (2x), trnI-GAU (2x), trnK-UUU, trnL-CAA (2x), trnL-UAA, trnL-UAG, trnM-CAU, trnN-GUU (2x), trnP-UGG, trnQ-UUG, trnR-ACG (2x), trnR-UCU, trnS-GCU, trnS-GGA, trnS-UGA, trnT-GGU, trnT-UGU, trnV-GAC (2x), trnV-UAC, trnW-CCA, trnY-GUA	trnA-UGC (2x), trnC-GCA, trnD-GUC, trnE-UUC, trnF-GAA, trnfM-CAU, trnG-UCC, trnH-GUG, trnI-CAU (2x), trnI-GAU (2x), trnK-UUU trnL-CAA(2x),,trnL-UAA ,trnL-UAG ,trnM-CAU ,trnN-GUU (2x), trnP-UGG ,trnQ-UUG ,trnR-ACG(2x) ,trnR-UCU ,trnS-GCU ,trnS-GGA ,trnS-UGA ,trnT-GGU ,trnT-UGU ,trnV-GAC (2x),trnV-UAC, trnW-CCA, trnY-GUA	trnA-UGC (2x),trnC-GCA,trnD-GUC,trnE-UUC,trnF-GAA,trnfM-CAU,trnG-UCC,trnH-GUG,trnI-CAU (2x), trnI-GAU(2x),trnK-UUU,trnL-CAA(2x),trnL-UAA,trnL-UAG,trnM-CAU,trnN-GUU (2x),trnP-UGG,trnQ-UUG,trnR-ACG(2x),trnR-UCU,trnS-GCU,trnS-GGA,trnS-UGA,trnT-GGU,trnT-UGU,trnV-GAC (2x),trnV-UAC,trnW-CCA,trnY-GUA	trnA-UGC (2x), trnC-GCA, trnD-GUC, trnE-UUC, trnF-GAA, trnfM-CAU, trnG-UCC, trnH-GUG, trnI-CAU (2x), trnI-GAU (2x), trnK-UUU, trnL-CAA (2x), trnL-UAA, trnL-UAG, trnM-CAU, trnN-GUU (2x), trnP-UGG, trnQ-UUG, trnR-ACG (2x), trnR-UCU, trnS-GCU, trnS-GGA, trnS-UGA, trnT-GGU, trnT-UGU, trnV-GAC (2x), trnV-UAC, trnW-CCA, trnY-GUA

Ribossomal RNAs (16S, 23S, 4.5S, 5S)	rrn16 (2x), rrn23 (2x), rrn4.5 (2x), rrn5 (2x)	rrn16 (2x), rrn23 (2x), rrn4.5 (2x), rrn5 (2x)	rrn16 (2x), rrn23 (2x), rrn4.5 (2x), rrn5 (2x)	rrn16 (2x), rrn23 (2x), rrn4.5 (2x), rrn5 (2x)	rrn16 (2x), rrn23 (2x), rrn4.5 (2x), rrn5 (2x)	rrn16 (2x), rrn23 (2x), rrn4.5 (2x), rrn5 (2x)	rrn16 (2x), rrn23 (2x), rrn4.5 (2x), rrn5 (2x)	rrn16 (2x), rrn23 (2x), rrn4.5 (2x), rrn5 (2x	rrn16 (2x), rrn23 (2x), rrn4.5 (2x), rrn5 (2x)
Ribossomal Protein small subunit	rps2, rps3, rps4, rps7 (2x), rps8, rps11, rps12 (2x), rps14, rps15, rps18, rps19	rps2, rps3, rps4, rps7 (2x), rps8, rps11, rps12 (2x), rps14, rps15, rps18, rps19	rps2, rps3, rps4, rps7 (2x), rps8, rps11, rps12 (2x), rps14, rps15, rps18, rps19	rps2, rps3, rps4, rps7 (2x), rps8, rps11, rps14, rps15, rps18, rps19	rps2, rps3, rps4, rps7 (2x), rps8, rps11, rps14, rps15, rps18, rps19	rps2, rps3, rps4, rps7 (2x), rps8, rps11, rps14, rps15, rps18, rps19	rps2, rps3, rps4, rps7 (2x), rps8, rps11,rps12(2x), rps14, rps15, rps18, rps19	rps2, rps3, rps4, rps7 (2x), rps8, rps11, rps12 (2x), rps14, rps15, rps18, rps19	rps2, rps3, rps4, rps7 (2x), rps8, rps11, rps14, rps15, rps18, rps19
Ribossomal Protein large subunit	rpl2, rpl14, rpl16, rpl20, rpl23 (2x), rpl32, rpl33, rpl36	rpl2, rpl14, rpl16, rpl20, rpl23 (2x), rpl32, rpl33, rpl36	rpl2, rpl14, rpl16, rpl20, rpl23 (2x), rpl32, rpl33, rpl36	rpl2 (2x), rpl14, rpl16, rpl20, rpl23 (2x), rpl32, rpl33, rpl36	rpl2 (2x), rpl14, rpl16, rpl20, rpl23 (2x), rpl32, rpl33, rpl36	rpl2 (2x), rpl14, rpl16, rpl20, rpl23 (2x), rpl32, rpl33, rpl36	rpl2, rpl14, rpl16, rpl20, rpl23 (2x), rpl32, rpl33, rpl36	rpl2, rpl14, rpl16, rpl20, rpl23 (2x), rpl32, rpl33, rpl36	rpl2, rpl14, rpl16, rpl20,rpl22, rpl23 (2x), rpl32, rpl33, rpl36
Subunits (α, β, β', β") of the DNA-dependent RNA polymerase	rpoA, rpoB, rpoC1, rpoC2	rpoA, rpoB, rpoC1, rpoC2	rpoA, rpoB, rpoC1, rpoC2	rpoA, rpoB, rpoC1, rpoC2	rpoA, rpoB, rpoC1, rpoC2	rpoA, rpoB, rpoC1, rpoC2	rpoA, rpoB, rpoC1, rpoC2	rpoA, rpoB, rpoC1, rpoC2	rpoA, rpoB, rpoC1, rpoC2
Photosystem I	psaA , psaB, psaC, psaI, psaJ	psaA , psaB, psaC, psaI, psaJ	psaA , psaB, psaC, psaI, psaJ	psaA, psaB, psaC, psaI, psaJ	psaA, psaB, psaC, psaI, psaJ	psaA, psaB, psaC, psaI, psaJ	psaA, psaB, psaC, psaI, psaJ	psaA, psaB, psaC, psaI, psaJ	psaA, psaB, psaC, psaI, psaJ
Photosystem II	psbA, psbB, psbC, psbD, psbE, psbF, psbH, psbI, psbJ, psbK, psbL, psbM, psbN, psbT, psbZ	psbA, psbB, psbC, psbD, psbE, psbF, psbH, psbI, psbJ, psbK, psbL, psbM, psbN, psbT, psbZ	psbA, psbB, psbC, psbD, psbE, psbF, psbH, psbI, psbJ, psbK, psbL, psbM, psbN, psbT, psbZ	psbA, psbB, psbC, psbD, psbE, psbF, psbH, psbI, psbJ, psbK, psbL, psbM, psbN, psbT, psbZ	psbA, psbB, psbC, psbD, psbE, psbF, psbH, psbI, psbJ, psbK, psbL, psbM, psbN, psbT, psbZ	psbA, psbB, psbC, psbD, psbE, psbF, psbH, psbI, psbJ, psbK, psbL, psbM, psbN, psbT, psbZ	psbA, psbB, psbC, psbD, psbE, psbF, psbH, psbI, psbJ, psbK, psbL, psbM, psbN, psbT, psbZ	psbA, psbB, psbC, psbD, psbE, psbF, psbH, psbI, psbJ, psbK, psbL, psbM, psbN, psbT, psbZ	psbA, psbB, psbC, psbD, psbE, psbF, psbH, psbI, psbJ, psbK, psbL, psbM, psbN, psbT, psbZ

	Cythochrome b/f complex	petA, petB, petD, petG, petL, petN	petA, petB, petD, petG, petL, petN	petA, petB, petD, petG, petL, petN	petA, petB, petD, petG, petL, petN	petA, petB, petD, petG, petL, petN	petA, petB, petD, petG, petL, petN	petA, petB, petD, petG, petL, petN	petA, petB, petD, petG, petL, petN	petA, petB, petD, petG, petL, petN
	ATP synthase	atpA, atpB, atpE, atpF, atpH, atpI	atpA, atpB, atpE, atpF, atpH, atpI	atpA, atpB, atpE, atpF, atpH, atpI	atpA, atpB, atpE, atpF, atpH, atpI	atpA, atpB, atpE, atpF, atpH, atpI	atpA, atpB, atpE, atpF, atpH, atpI	atpA, atpB, atpE, atpF, atpH, atpI	atpA, atpB, atpE, atpF, atpH, atpI	atpA, atpB, atpE, atpF, atpH, atpI
**Photosynthesis**	NADH-dehydrogenase	ndhA, ndhB (2×), ndhC, ndhD, ndhE, ndhF, ndhG, ndhH, ndhI, ndhJ, ndhK	ndhA, ndhB (2×), ndhC, ndhD, ndhE, ndhF, ndhG, ndhH, ndhI, ndhJ, ndhK	ndhA, ndhB (2×), ndhC, ndhD, ndhE, ndhF, ndhG, ndhH, ndhI, ndhJ, ndhK	ndhA, ndhB (2x), ndhC, ndhD, ndhE, ndhF, ndhG, ndhH, ndhI, ndhJ, ndhK	ndhA, ndhB (2x), ndhC, ndhD, ndhE, ndhF, ndhG, ndhH, ndhI, ndhJ, ndhK	ndhA, ndhB (2x), ndhC, ndhD, ndhE, ndhF, ndhG, ndhH, ndhI, ndhJ, ndhK	ndhA, ndhB (2x), ndhC, ndhD, ndhE, ndhF, ndhG, ndhH, ndhI, ndhJ, ndhK	ndhA, ndhB (2x), ndhC, ndhD, ndhE, ndhF, ndhG, ndhH, ndhI, ndhJ, ndhK	ndhA, ndhB (2x), ndhC, ndhD, ndhE, ndhF, ndhG, ndhH, ndhI, ndhJ, ndhK
	Large subunit RUBISCO	rbcL	rbcL	rbcL	rbcL	rbcL	rbcL	rbcL	rbcL	rbcL
	Acetyl-CoA carboxylase	accD	accD	accD	accD	accD	accD	accD	accD	accD
	Cythochrome c biogenesis	ccsA	ccsA	ccsA	ccsA	ccsA	ccsA	ccsA	ccsA	ccsA
	Maturase	matK	matK	matK	matK	matK	matK	matK	matK	matK
**Miscellaneous**	ATP-dependent protease	clpP	clpP	clpP	clpP	clpP	clpP	clpP	clpP	clpP
	Inner membrane protein	cemA	cemA	cemA	cemA	cemA	cemA	cemA	cemA	cemA
**Pseudogene unknown function**	Conserved hypothetical chloroplast ORFs	ycf1, ycf2 (2×), ycf3, ycf4	ycf1, ycf2 (2×), ycf3, ycf4	ycf1, ycf2 (2×), ycf3, ycf4	ycf1 (3x), ycf2 (2x), ycf3, ycf4	ycf1 (3x), ycf2 (2x), ycf3, ycf4	ycf1 (3x), ycf2 (2x), ycf3, ycf4	ycf1 (2x), ycf2 (2x), ycf3, ycf4	ycf1 (2x), ycf2 (2x), ycf3, ycf4	ycf1 (2x), ycf2 (2x), ycf3, ycf4
