# Supplementary material for: High-Throughput Genomic Data Reveal Complex Phylogenetic Relationships in Stylosanthes Sw (Leguminosae)
Source: Front Genet. 2021 Sep 23;12:727314. doi: 10.3389/fgene.2021.727314 (PMC8495327; doi:10.3389/fgene.2021.727314)
Supplement: Supplementary file 2 [file DataSheet1.PDF]

Supplementary Figures

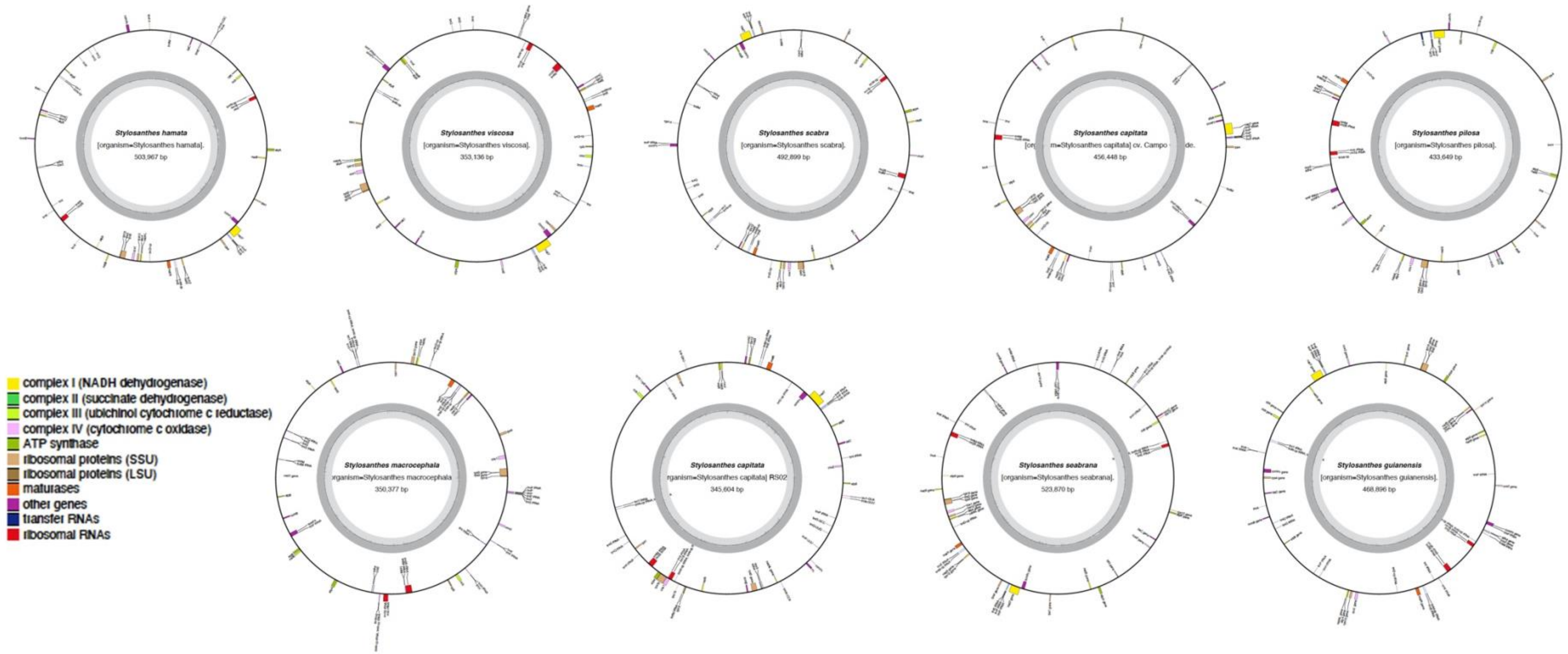

Supplementary Figure 1. Circular gene maps of newly generated mitogenomes for *Stylosanthes hamata*, *S. viscosa*, *S. scabra*, *S. capitata* cv. Campo Grande, *S. pilosa*, *S. macrocephala*, *S. capitata* RS024, *S. seabrana* and *S. guianensis*. Genes on the inside of outer circles are transcribed in a clockwise direction, while genes on the outside of outer circles are transcribed in a reverse direction.

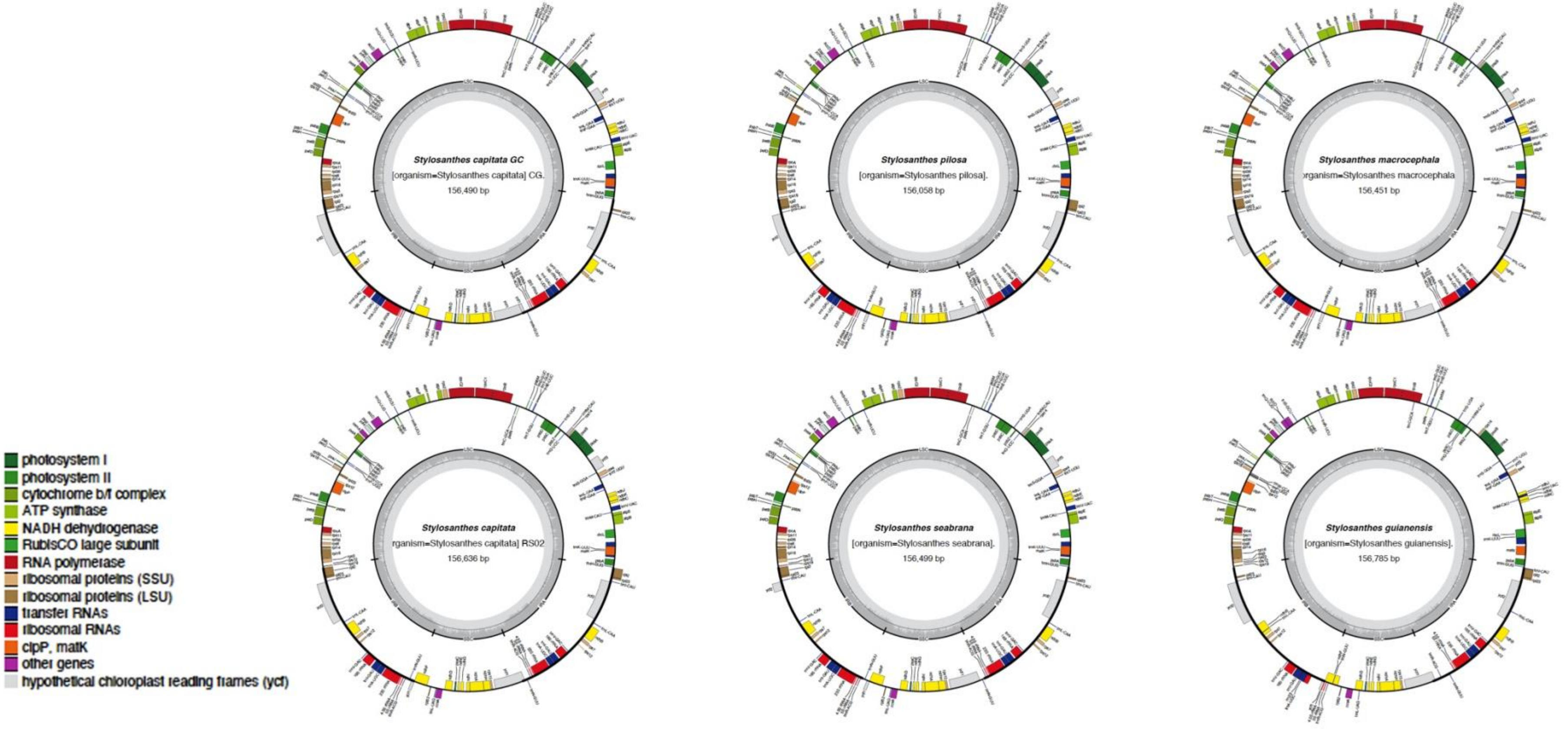

Supplementary Figure 2. Circular gene maps of newly generated plastomes for *S. capitata* cv. Campo Grande, *S. pilosa*, *S. macrocephala*, *S. capitata* RS024, *S. seabrana* and *S. guianensis*. Genic groups are related with colors. Limits of regions LSC, IRa, IRb and SSC are indicated in the inner circle.

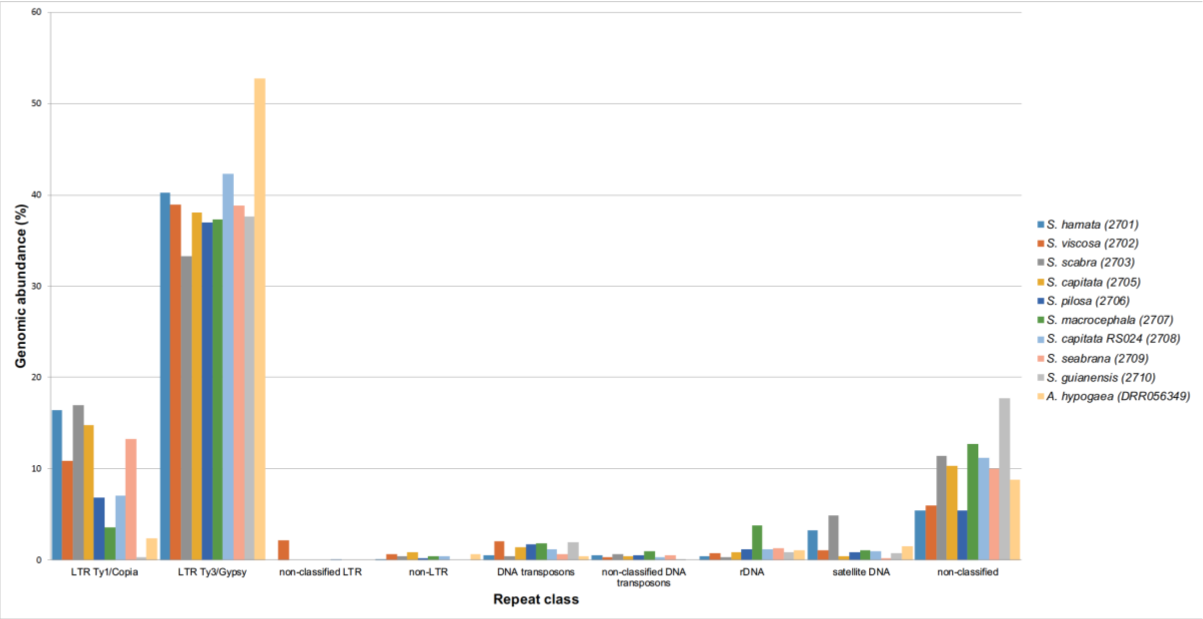

Supplementary Figure 3. Comparative analysis of the main classes of repeats in *Stylosanthes*.

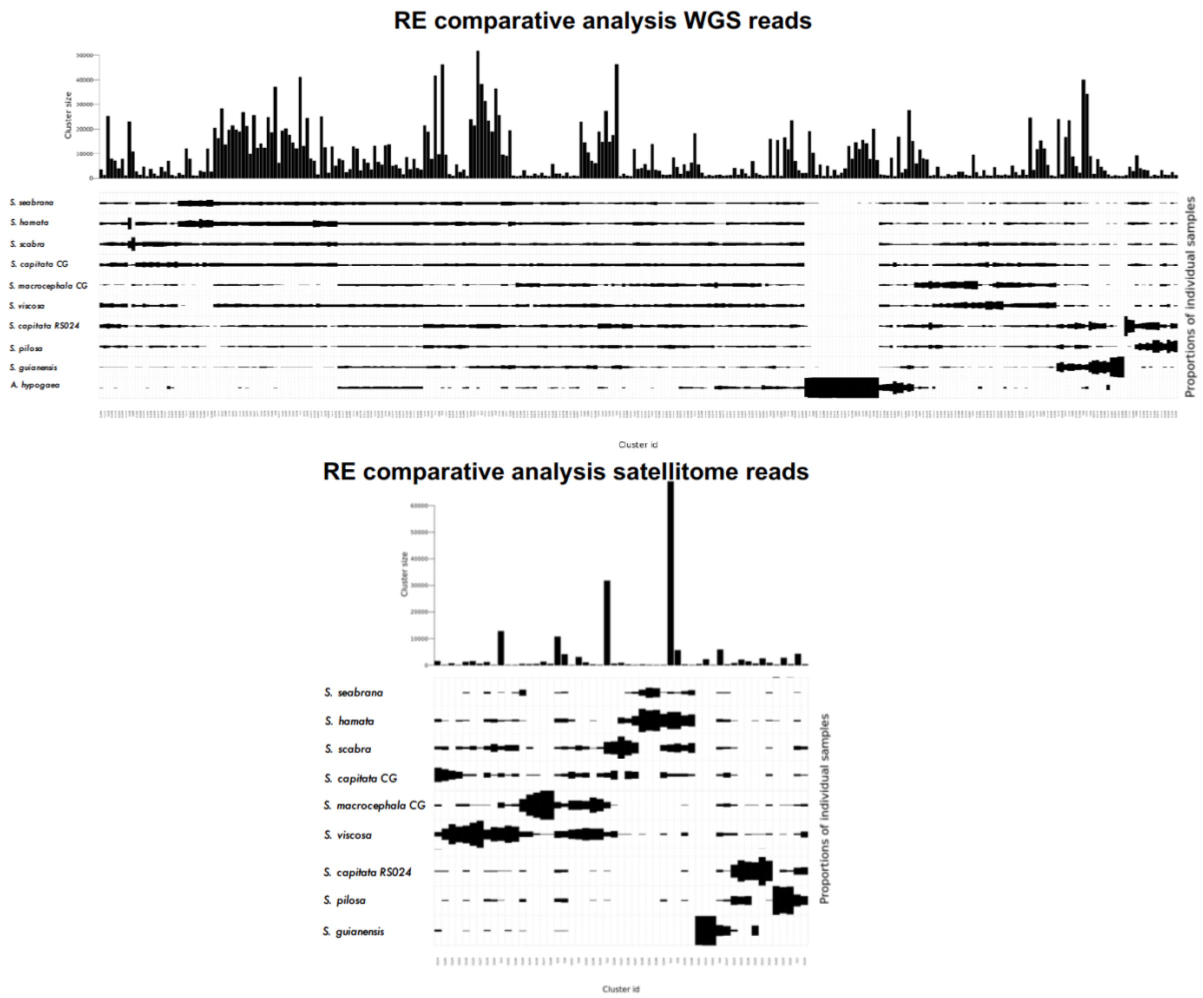

Supplementary Figure 4. Comparative RE2 analysis showing the cumulative abundance across each repeat cluster. Note that both total repeat and satellitome abundance matches species phylogenetic relationships.

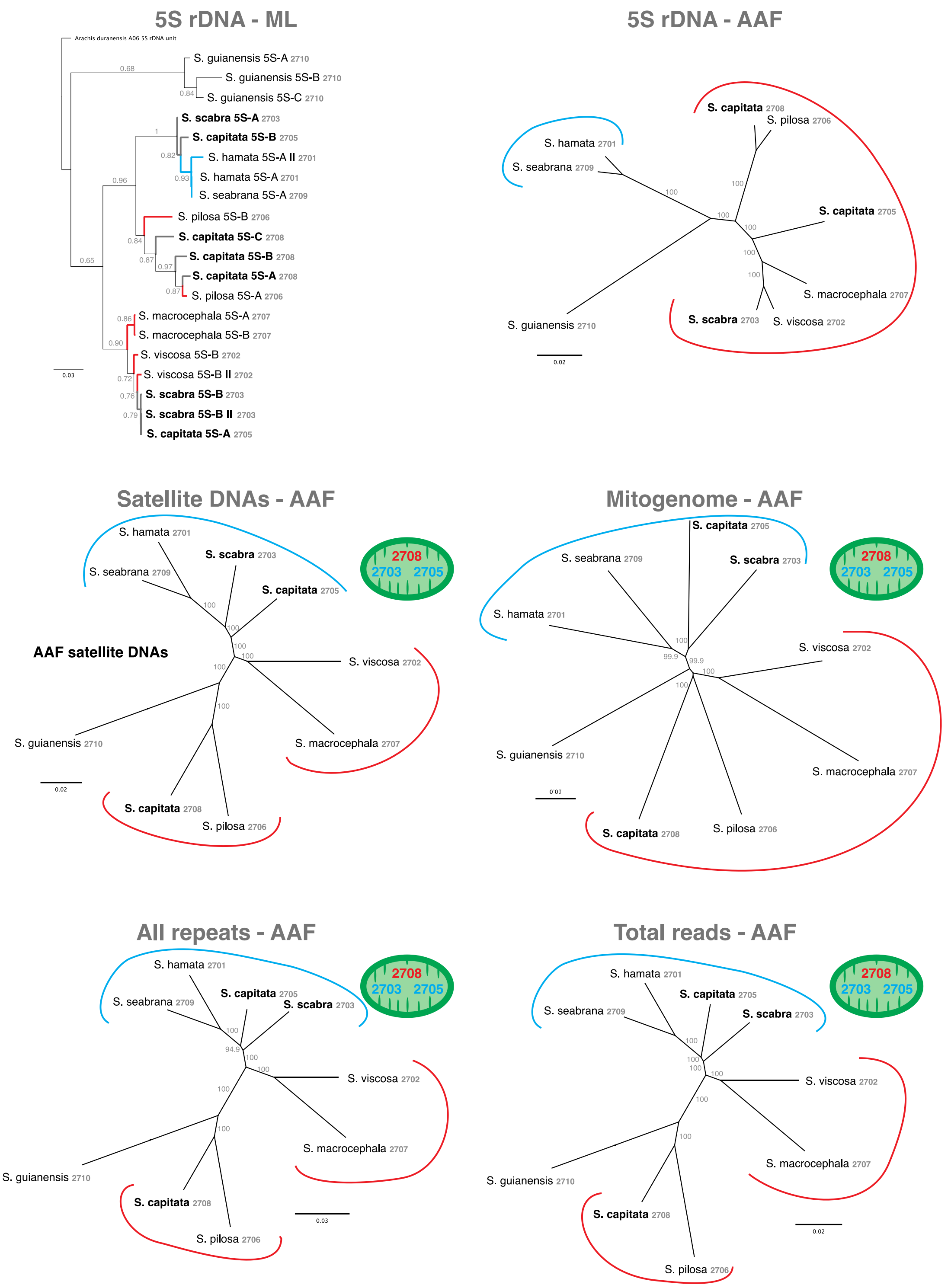

Supplementary Figure 5. Alignment-based and AFF phylogenies of different datasets including *Stylosanthes* allopolyploids.

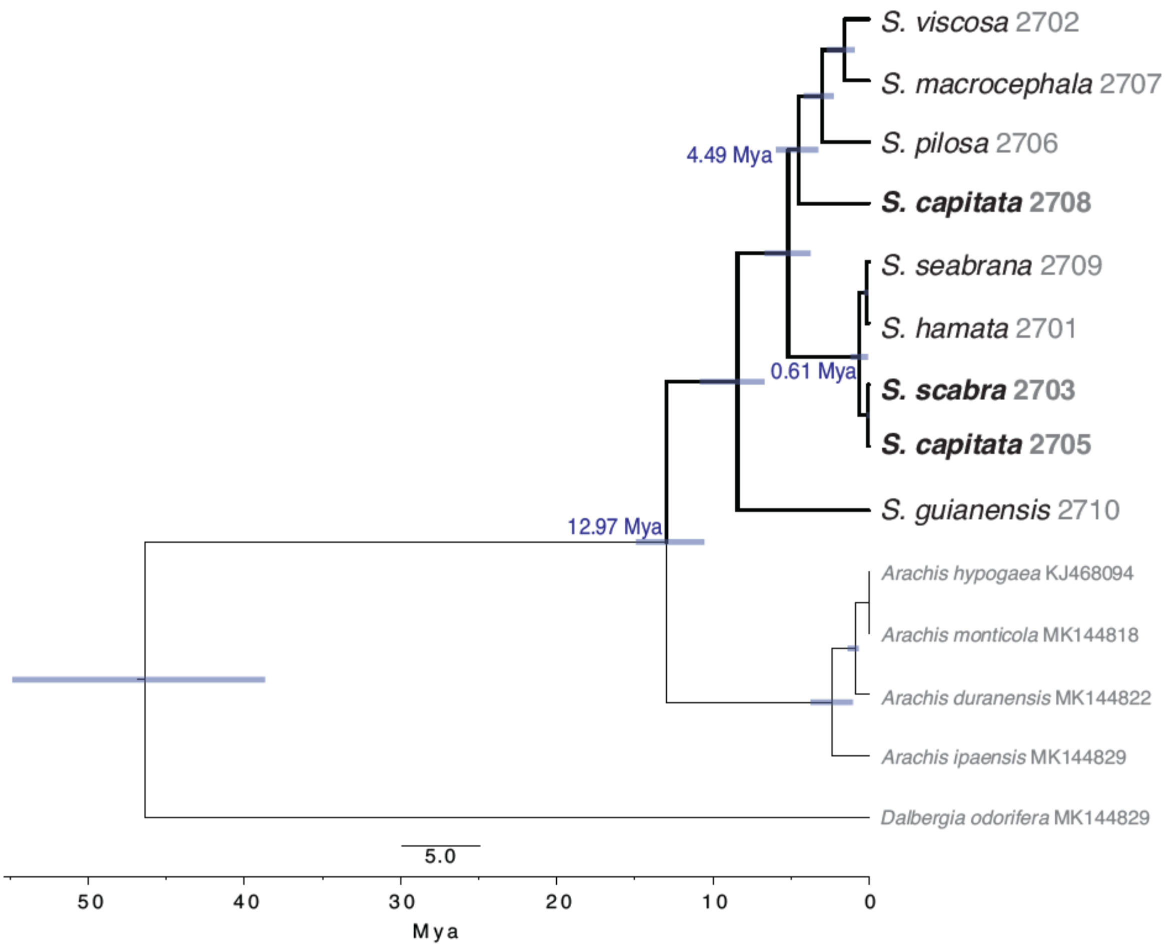

Supplementary Figure 6. A dated Bayesian phylogeny of Stylosanthes showing the estimated origin times for allopolyploids.

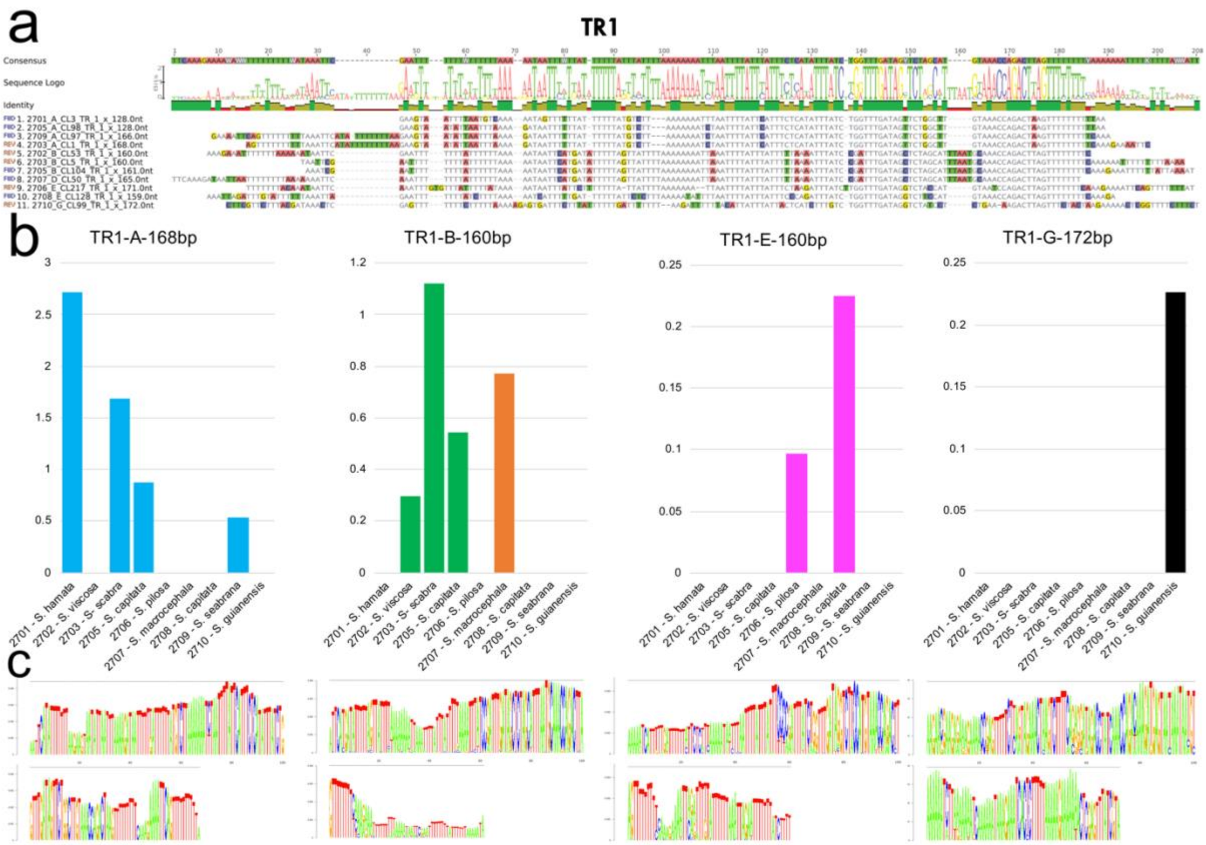

Supplementary Figure 7. Characterization of TR1, the main family of satellite DNA in *Stylosanthes*. (a) Alignment of the consensus sequences of TR1 extracted from all *Stylosanthes* species analyzed. (b) Genomic abundance of each TR1 variant across all *Stylosanthes* species. (c) Sequence logo of each TR1 variant showing the base-per-base sequence variability found.
